# Statistical significance approximation for local similarity analysis of dependent time series data

**DOI:** 10.1186/s12859-019-2595-x

**Published:** 2019-01-28

**Authors:** Fang Zhang, Fengzhu Sun, Yihui Luan

**Affiliations:** 1 School of Mathematics, Shandong University, Jinan, Shandong, 250100 China; 2 Quantitative and Computational Biology Program, Department of Biological Sciences, University of Southern California, 1050 Childs Way, Los Angeles, 90089 CA USA; 3 Institute of Science and Technology for Brain-inspired Intelligence, Fudan University, Shanghai, 200433 China

**Keywords:** Data-driven local similarity analysis, Long-run variance, Nonparametric kernel estimate, Statistical significance

## Abstract

**Background:**

Local similarity analysis (LSA) of time series data has been extensively used to investigate the dynamics of biological systems in a wide range of environments. Recently, a theoretical method was proposed to approximately calculate the statistical significance of local similarity (LS) scores. However, the method assumes that the time series data are independent identically distributed, which can be violated in many problems.

**Results:**

In this paper, we develop a novel approach to accurately approximate statistical significance of LSA for dependent time series data using nonparametric kernel estimated long-run variance. We also investigate an alternative method for LSA statistical significance approximation by computing the local similarity score of the residuals based on a predefined statistical model. We show by simulations that both methods have controllable type I errors for dependent time series, while other approaches for statistical significance can be grossly oversized. We apply both methods to human and marine microbial datasets, where most of possible significant associations are captured and false positives are efficiently controlled.

**Conclusions:**

Our methods provide fast and effective approaches for evaluating statistical significance of dependent time series data with controllable type I error. They can be applied to a variety of time series data to reveal inherent relationships among the different factors.

**Electronic supplementary material:**

The online version of this article (10.1186/s12859-019-2595-x) contains supplementary material, which is available to authorized users.

## Background

Next generation sequencing (NGS) technologies have made it possible to generate a large amount of time series data in both genomics and metagenomics. An important question in time series data analysis is the identification of associated factors, where the factors can be genes in gene expression analysis or operational taxonomic units (OTUs) in metagenomic studies. Specifically, the abundance series of OTUs are used to investigate the temporal variation of microbial communities in longitudinal studies [1]. Most commonly used approaches for identifying associated factors are to calculate the Pearson correlation coefficients (PCC) or Spearman correlation coefficients (SPCC) among the factors and to identify the significantly associated pairs of factors. However, it was observed in previous studies that factors can be associated in a subset of time intervals (local) and maybe there are time-delays between the factors. PCC and SPCC may fail to identify such local associations with/without time-delays.

Several methods have been developed to understand such associations and have been applied to analyze gene expression profiles [2–4], regulatory network construction [5], co-occurrence patterns in microbial communities [6–9] and many other fields [10, 11]. For example, Qian et al. [2] proposed a local similarity method to identify potential local and time-shift relationships between gene expression data. Ji and Tan [4] suggested a similar procedure that switched gene expression profiles into distinctive changing trend states and calculated the local similarity of the new time series. Ruan et al. [7] investigated local relationships among microbial organisms and environment factors in the San Pedro Channel in the North Pacific Ocean and visualized the graphical structure of significant local similarity associations. Xia et al. [11] extended this method to investigate the replicated time series data and obtained confidence interval of LSA by bootstrap. In these studies, permutation test was used to evaluate statistical significance of the local similarity score, which is time-consuming if a large number of factors are considered.

To overcome the computational issues of permutation test, several research groups developed theoretical approaches to approximate the statistical significance of LSA [12–14]. However, both permutation test and the theoretical approximations require the assumption that the time series are independent identical distributed (i.i.d.), which can be violated in most time series data.

In this study, we develop two new methods, referred to as data-driven LSA (DDLSA) and LSA for residues (LSAres), to more accurately approximate the statistical significance of LSA. DDLSA employs long-run covariance (described below) of stationary time series through nonparametric kernel estimate to evaluate statistical significance of the original LSA, while LSAres uses the residuals from a predefined model as a substitute for the original series to calculate the statistical significance, similar to the idea of local trend analysis [14]. We investigate the size and power of different approaches and show the validity of our methods using simulations. Further, we apply these methods to analyze human microbiome and marine microbial communities from different high-throughput experiments and compare the identified associated factors using our newly developed methods and those from previous theoretical approximations of LSA scores.

## Methods

In this section, we first present an outline of the definition of LSA as given in [2, 7] and the theoretical approximation of statistical significance of the LSA score in [12]. Second, we present our new data driven LSA (DDLSA) approach for evaluating statistical significance of LSA for dependent time series data. For easy reading, the details of the methods are given as additional information. Third, we present the simulation strategies to evaluate the size and power of the different approaches. Fourth, we describe the human and marine metagenomic data used to demonstrate the applications of our new approaches.

### Outline of LSA and theoretical approximation of statistical significance

Consider two time series *X*_*t*_ and *Y*_*t*_,*t*=1,⋯,*n*, with mean 0. The local similarity analysis [2, 7] was developed to find intervals of the same length from each sequence to maximize the similarity between the two time series. In practice, biologists are only interested in a relatively small number of delays. Therefore, it is required that the starting positions of the intervals differ by at most *D*, a parameter set by the practitioners. A dynamic programming algorithm was developed to calculate the largest similarity score, referred to as local similarity (LS) score. The idea was very similar to local sequence alignment in molecular sequence analysis [15]. In these early studies, statistical significance of the LS score was evaluated using permutations. Particularly, one of the time series data was fixed and the other one was permutated many times, and the resulting LS score was obtained using the dynamic programming algorithm. The *p*-value was approximated by the fraction of times the LS score of the permuted data is larger than the LS score of the actual data.

There are several drawbacks to permutation test for approximating the statistical significance of the LS score. On the one hand, permutation test requires that data is independent at different time points. However, in practical problems, this assumption is usually violated and time series data may depend on the values of the previous time points. On the other hand, the permutation procedure is time-consuming, especially when the *p*-value precision is small, as the time complexity is inversely proportional to the *p*-value precision. When the number of factors is large, all pairwise analysis of high-throughput data is computationally challenging. Therefore, fast and efficient methods to obtain statistical significance approximation of LS score are needed.

Xia et al. [12] and Durno et al. [13] independently developed theoretical approximations for the *p*-value. Let *s*_*D*_ be the LS score with maximum delay of *D* between *X*_*t*_ and *Y*_*t*_. Xia et al. [12] approximated the *p*-value by $\mathcal {L}_{D}\left (s_{D}/(\sigma \sqrt {n})\right) $, where

1 $$ \begin{aligned} &\mathcal{L}_{D}(x) \approx 1-8^{2D+1}\\&\left[\sum_{k=1}^{\infty}\left\{\frac{1}{x^{2}}+\frac{1}{(2k-1)^{2}\pi^{2}}\right\}\exp\left\{-\frac{(2k-1)^{2}\pi^{2}}{2x^{2}}\right\}\right]^{2D+1}, \end{aligned}  $$

and *n* is the number of time points. If both *X*_*t*_ and *Y*_*t*_ are i.i.d, *σ*^2^=*var*(*X*_*t*_*Y*_*t*_). If both *X*_*t*_ and *Y*_*t*_ are first order Markov chains (such as DNA sequences in the identification of CpG islands [16]), $\sigma ^{2} = E_{\phi }\left (Z_{1}^{2}\right) + 2\sum _{k=1}^{\infty }E_{\phi }(Z_{1}Z_{k+1})$, with *Z*_*t*_=*X*_*t*_*Y*_*t*_. Details on these approximations are given in Additional file 1.

### Statistical significance of LS score for dependent time series

Time series data in general depend on each other and cannot be best modelled by Markov chains. Moreover, it is challenging to obtain *σ*^2^ defined above for Markov models. Therefore, we provide a data driven approach for evaluating the statistical significance of LS score for dependent time series data.

Assume *X*_*t*_ and *Y*_*t*_ are weakly stationary time series with mean 0. Here a time series *X*_*t*_ is weakly stationary if *E*|*X*_*t*_|^2^<*∞*, *E*(*X*_*t*_) is a constant (independent of *t*) and *Cov*(*X*_*t*_,*X*_*t*+*k*_) depends only on time delay *k*. Under the null hypothesis *H*_0_ that the two time series are not associated, *Z*_*t*_=*X*_*t*_*Y*_*t*_ is also weakly stationary with mean 0. Using similar arguments as in [12], we can show that the *p*-value can again be approximated by $\mathcal {L}_{D}\left (s_{D}/(\omega \sqrt {n})\right) $, where the function $\mathcal {L}_{D}$ is given in Eq. 1 and $\omega ={\lim }_{n \rightarrow \infty }\sqrt {var\left (\sum _{i=1}^{n}Z_{i}\right)/n}$ is referred to as the long-run variance. The details of theoretical derivations are given in the Additional file 1.

The estimate of *ω* plays a crucial role in deriving the statistical significance of LS score and has an enormous impact on the validity of local similarity analysis for dependent data. Following Andrew [17], we used an autoregressive (AR)(1) plug-in data dependent method to estimate the long-run variance. The autoregressive model specifies that the current value depends linearly on its own previous values.

Let $\hat {\gamma }_{z}(k)$ be the sample autocovariance function of *Z*_*t*_, defined as:

2 $$ \hat{\gamma}_{z}(k)=\frac{1}{n}\sum\limits_{j=1}^{n-|k|}\left(Z_{j}-\bar{Z}\right)\left(Z_{j+|k|}-\bar{Z}\right), k=1,2,\cdots,n-1,  $$

where $\bar {Z}=\frac {1}{n}\sum _{i=1}^{n}Z_{i}$ is the mean of *Z*_*t*_. Under the null hypothesis *H*_0_, we can approximate $\hat {\gamma }_{z}(k)$ by $\hat {\gamma }_{x}(k) \hat {\gamma }_{y}(k)$ if the means of *X*_*t*_ and *Y*_*t*_ are zero, where $\hat {\gamma _{x}}(k)$, $\hat {\gamma _{y}}(k)$ and $\hat {\gamma _{z}}(k)$ are the sample autocovariance functions of *X*_*t*_, *Y*_*t*_ and *Z*_*t*_, respectively. We can estimate *ω* by

3 $$ \hat{\omega}_{n}^{2}=\hat{\gamma}_{x}(0)\hat{\gamma}_{y}(0)+2\sum\limits_{k=1}^{b_{w}}\left(1-\frac{k}{b_{w}}\right)\hat{\gamma}_{x}(k)\hat{\gamma}_{y}(k),  $$

where *b*_*ω*_ is the bandwidth parameter $b_{\omega }=\left \lfloor 1.1447(\hat {\tau }n)^{1/3}\right \rfloor $ [17],

4 $$ \hat{\tau}=\frac{4\hat{\phi}^{2}}{\left(1-\hat{\phi}^{2}\right)^{2}}, \qquad \hat{\phi}=\frac{\sum\limits_{i=2}^{n}\hat{u}_{t}\hat{u}_{t-1}}{\sum\limits_{i=2}^{n}\hat{u}_{t}^{2}}, \qquad \hat{u}_{t}=Z_{t}-\bar{Z}.  $$

In summary, given time series *X*_*t*_ and *Y*_*t*_, we first calculate their LS score *s*_*D*_ using the dynamic programming algorithm in [7]. We then estimate the long-run variance using Eq. 3. Finally, the statistical significance of the LS score for dependent data can be approximated as $\mathcal {L}_{D}(s_{D}/(\hat {\omega }_{n}\sqrt {n}))$. Since we estimate the long-run variance from real data, we refer to the new method as data driven LSA (DDLSA).

### Local similarity analysis based on residuals

We also modified the original theoretical approximation of statistical significance of LS score [12] by considering the residuals of the original time series. First we suppose that time series data are generated from a pre-defined model, such as autoregressive (AR) model or autoregressive moving average (ARMA) model. We then use the residuals from the model as the substitution of the original data, since the correlation of data may come from the relevance of the residuals. Because of the independent property of the residuals, the statistical significance of LS score of residuals can be obtained from the approximate theoretical distribution of LSA for i.i.d. time series (Eq. 1). We refer to this method as LSAres.

### Simulation studies

We evaluated the size and power of six different methods for determining the statistical significance of associations between factors in time series data. The six methods are described as follows.

1. **PCC**. Pearson correlation coefficient (PCC) is widely used to identify correlation between random variables. If the random variables *X*_*t*_ and *Y*_*t*_ are from a bivariate normal distribution and their PCC is *r*, the statistic $t=r\sqrt {(n-2)/(1-r^{2})}$ has a Student’s t-distribution with degrees of freedom *n*−2 under the null hypothesis *H*_0_.
2. **SRCC**. Spearman rank correlation coefficient (SRCC, *r*_*s*_) between *X*_*t*_ and *Y*_*t*_ is defined as Pearson correlation coefficient between the rank values of those two variables. We can test for the significance of *r*_*s*_ using $t=r_{s}\sqrt {(n-2)/\left (1-r_{s}^{2}\right)}$, which follows approximately a Student t-distribution with degrees of freedom *n*−2.
3. **Theoretical LSA (TLSA)**. We used the procedures in [12] to calculate the *p*-value of the LS score between *X*_*t*_ and *Y*_*t*_.
4. **Permutation test**. We fixed one time series *Y*_*t*_ and reshuffled *X*_*t*_ for *N*=1000 times. Assuming that $X_{t}^{(k)}, k=1,\cdots,N$ were the permutations of *X*_*t*_, we computed the LS score between $X_{t}^{(k)}$ and *Y*_*t*_, denoted as $s_{D}^{(k)}$. Then the *p*-value was approximated by the fraction of times that $s_{D}^{(k)}$ are at least as high as *s*_*D*_, the LS score between *X*_*t*_ and *Y*_*t*_.
5. **LSAres**. We adopted the AR or ARMA models to obtain the residuals of data, and calculated the statistical significance of the residuals through TLSA, which was regarded as the significance between *X*_*t*_ and *Y*_*t*_.
6. **DDLSA**. In DDLSA, the time series data need to be centered first. Specifically, time series data *X*_*t*_,*t*=1,2,⋯,*n* are centered as $\tilde {X}_{t} = X_{t} - \bar {X}_{t}$, where $\bar {X}_{t} = \frac {1}{n}\sum _{t=1}^{n}X_{t}$ is the sample mean of *X*_*t*_. $\tilde {Y}_{t}$ is defined analogously. We utilized $\mathcal {L}_{D}\left (s_{D}/\left (\hat {\omega }_{n}\sqrt {n}\right)\right)$ to calculate the approximate statistical significance of $\tilde {X}_{t}$ and $\tilde {Y}_{t}$ and took it as the significance between *X*_*t*_ and *Y*_*t*_.

#### Comparison of the empirical size of different approaches

We investigated whether *p*-values obtained from these statistics were close to the significance level which is the probability rejecting the null hypothesis, given that it were true. Here we used three different null models to compare the size of the six approaches for calculating the statistical significance of the LS score:

1. **The AR(1) model:**
  5 $$ \begin{aligned} & X_{t}=\rho_{1}X_{t-1}+\varepsilon_{t}^{X} \\ & Y_{t}=\rho_{2}Y_{t-1}+\varepsilon_{t}^{Y} \end{aligned}  $$
2. **The ARMA(1,1) model:**
  6 $$ \begin{aligned} & X_{t}=\rho_{1}X_{t-1}+\varepsilon_{t}^{X}+0.5~\varepsilon_{t-1}^{X} \\ & Y_{t}=\rho_{2}Y_{t-1}+\varepsilon_{t}^{Y}+0.5~\varepsilon_{t-1}^{Y} \end{aligned}  $$
3. **The ARMA(1,1)-TAR(1) model:**
  7 $$ \begin{aligned} & X_{t}=\rho_{1}X_{t-1}+\varepsilon_{t}^{X}+0.5~\varepsilon_{t-1}^{X} \\ & Y_{t}=\left\{\begin{array}{ll} \rho_{2}Y_{t-1}+\varepsilon_{t}^{Y}, Y_{t-1}\leq -1\\ 0.5~Y_{t-1}+\varepsilon_{t}^{Y}, Y_{t-1}> -1 \end{array}\right. \end{aligned}  $$

where 0<|*ρ*_1_|,|*ρ*_2_|<1, $\varepsilon _{t}^{X}$ and $\varepsilon _{t}^{Y}$ are independent standard normal random variables. All these models were stationary. For each model, we first generated *X*_0_ and *Y*_0_ from the standard normal distribution. Then we generated (*X*_*t*_,*Y*_*t*_), *t*=2,⋯,100+*n* from these models. Finally, we discarded the first 100 samples and took the others as the true *X*_*t*_ and *Y*_*t*_. The procedure can guarantee the stationarity of the time series generated from these models.

#### Comparison of the empirical power of different approaches

Next we investigated the power of the six methods for detecting the association between the factors under two alternative models that the factors are associated. Our objective is to identify the most powerful method for detecting the associations between the factors.

**Fig. 1:**
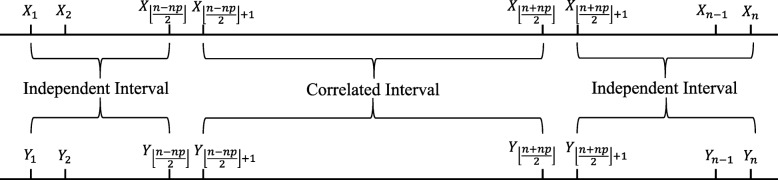
Diagrammatic sketch of data generating process in the local and bivariate AR models. The middle intervals of *X*_*t*_ and *Y*_*t*_ are correlated and both ends of them are independent. Here ⌊·⌋ is the floor function which returns the greatest integer less than or equal to the input

**The local AR model** We studied a model that the two factors are only associated in a subinterval:

8 $$ \begin{aligned} X_{1} = \varepsilon_{1}^{X}, ~~~X_{t} &= \rho_{1}X_{t-1}+\varepsilon_{t}^{X}, t=2,\cdots,n, \\ Y_{1} =\varepsilon_{1}^{Y}, ~~~Y_{t} &= \rho_{2}Y_{t-1}+\varepsilon_{t}^{Y}, t=2,\cdots,n, \end{aligned}  $$

where $\varepsilon _{1}^{X},\varepsilon _{1}^{Y}\sim N(0,1),\varepsilon _{t}^{X}\sim N\left (0,1-\rho _{1}^{2}\right), \varepsilon _{t}^{Y}\sim N\left (0,1-\rho _{2}^{2}\right), t=2,\cdots,n$ and they are independent. For simplicity and symmetry, we generated time series data that were correlated within the middle interval of length *np* as follows, where *p* is the fraction of the time interval that the two time series were correlated (shown in Fig. 1). We first generated *X*_*t*_ using Eq. 8. Second, let $Y_{t}=\frac {1}{\sqrt {1+\sigma ^{2}}}(X_{t}+\xi _{t})$ in the middle *np* time points of the entire series where *ξ*_*t*_∼*N*(0,*σ*^2^),*σ*^2^=(1−*ρ*^2^)/*ρ*^2^. In the remaining *n*−*np* time points, *Y*_*t*_ were generated by the AR(1) model (Eq. 8) with *ρ*_2_=*ρ*_1_/(1+*σ*^2^). We generated the time series data this way so that *X*_*t*_ followed a stationary AR(1) model, *Y*_*t*_ approximately followed a stationary AR(1) model, and *X*_*t*_ and *Y*_*t*_ were correlated in the middle *np* time points with correlation coefficient *ρ*.

**The bivariate AR model** We also investigated another model, referred to as the bivariate AR(1) model, that was used in [18] (Chapter 7, page 290).

9 $$ \begin{aligned} X_{1} = \varepsilon_{1}^{X}, ~~~ X_{t} &= \rho_{1}X_{t-1}+\varepsilon_{t}^{X}, t=2,\cdots,n, \\ Y_{1} = \varepsilon_{1}^{Y}, ~~~Y_{t} &= \rho_{2}Y_{t-1}+\varepsilon_{t}^{Y}, t=2,\cdots,n, \end{aligned}  $$

where $\varepsilon _{1}^{X}, \varepsilon _{1}^{Y}\sim N(0,1), \varepsilon _{t}^{X}\sim N\left (0,1-\rho _{1}^{2}\right), \varepsilon _{t}^{Y}\sim N\left (0,1-\rho _{2}^{2}\right), t=2,\cdots,n$ and the noise terms have correlation coefficients:

10 $$ \begin{aligned} cor\left(\varepsilon_{1}^{X},\varepsilon_{1}^{Y}\right) &=\rho,\\ cor\left(\varepsilon_{t}^{X},\varepsilon_{t}^{Y}\right) &=\frac{(1-\rho_{1}\rho_{2})\rho}{\sqrt{\left(1-\rho_{1}^{2}\right)\left(1-\rho_{2}^{2}\right)}}, t=2,\cdots,n, \\ cor\left(\varepsilon_{i}^{X},\varepsilon_{j}^{Y}\right) &=0, i,j=1,\cdots,n, i \neq j. \end{aligned}  $$

The variances of both *X*_*t*_ and *Y*_*t*_ are 1 and *cor*(*X*_*t*_,*Y*_*t*_)=*ρ*. Similarly as above, we generated locally associated time series data. In the middle *np* time points, we generated (*X*_*t*_,*Y*_*t*_) using Eq. 9. In the remaining *n*−*np* time points, we generated (*X*_*t*_,*Y*_*t*_) by the independent bivariate AR(1) model with *ρ*=0.

### Applications to a human and a marine microbiome data sets

We applied DDLSA and LSAres to analyze a human and a marine microbiome time series data sets. The Moving Pictures of the Human Microbiome (MPHM) data was collected from two healthy subjects, one male (‘M3’) and one female (‘F4’). Both individuals were sampled daily at three body sites: gut (feces), mouth(tongue), and skin (left and right palms) [19]. The data set consists of 130, 135 and 133 daily samples from ‘F4’, and 332, 372 and 357 samples from ‘M3’. There are 335, 373 and 1295 operational taxonomic units (OTUs) from feces, tongue and palm (both left and right) sites of ‘F4’ and ‘M3’, where the taxonomic level is Genus. We selected 41 ‘core’ OTUs that were observed in at least 60% samples from the tongue of ‘F4’ and analyzed their relationships.

The PML data set is one of the longest microbial time series consisting of monthly samples taken over 6 years at a temperate marine coastal site off Plymouth, UK [20]. These samples were sequenced using high-resolution 16S rRNA tag NGS sequencing. A total of 155 bacterial OTUs were identified with the taxonomic level of Order. Among them, we chose 62 abundant OTUs that were present in at least 50% of the time points, and 13 environment factors to analyze their association network. We filled the missing values in the environment data using linear interpolation.

## Results and discussion

### DDLSA and LSAres have controlled type I error rates and other approaches do not

We investigated the effects of the autoregressive coefficients *ρ*_1_ and *ρ*_2_ and the number of time points *n* on the type I error rates of the six methods for evaluating statistical significance under the AR(1) (Eq. 5), ARMA(1,1) (Eq. 6) and ARMA(1,1)-TAR(1) (Eq. 7) models. We chose six different pairs of autoregressive coefficients from -0.5 to 0.8 and the number of time points *n* from 100 to 1000. The results are shown in Tables 1, 2 and 3 for the three models, respectively. For TLSA, Permutation test, LSAres and DDLSA, we set the maximum time delay *D*=0 for simplicity. For LSAres, we needed to specify the generative models for *X*_*t*_ and *Y*_*t*_. For given data, the generative models are most likely unknown. We used AR or ARMA models as generative models and denoted the resulting methods as LSAres(AR) and LSAres(ARMA), respectively. Throughout the simulations, we let the pre-specified error rate to be 0.05.

**Table 1:** The empirical type I error rates for the six different methods (the third to ninth column): PCC, SRCC, TLSA, permutation, LSAres(AR), LSAres(ARMA), and DDLSA, based on the AR(1) model

*ρ*_1_,*ρ*_2_	n	PCC	SRCC	TLSA	Permutation	LSAres(AR)	LSAres(ARMA)	DDLSA
-0.5 -0.5	100	0.1315	0.1261	0.1183	0.1647	0.0302	0.0324	0.0407
	200	0.1250	0.1216	0.1387	0.1714	0.0319	0.0359	0.0454
	300	0.1321	0.1282	0.1498	0.1768	0.0400	0.0378	0.0526
	500	0.1270	0.1209	0.1573	0.1809	0.0406	0.0359	0.0485
	1000	0.1233	0.1144	0.1703	0.1908	0.0387	0.0455	0.0509
0 0	100	0.0460	0.0459	0.0296	0.0477	0.0289	0.0312	0.0303
	200	0.0503	0.0501	0.0340	0.0485	0.0349	0.0319	0.0350
	300	0.0500	0.0516	0.0353	0.0483	0.0365	0.0386	0.0366
	500	0.0493	0.0502	0.0403	0.0502	0.0413	0.0388	0.0404
	1000	0.0484	0.0487	0.0434	0.0504	0.0429	0.0471	0.0441
0.3 0.3	100	0.0725	0.0716	0.0533	0.0814	0.0271	0.0281	0.0411
	200	0.0699	0.0691	0.0615	0.0824	0.0335	0.0371	0.0459
	300	0.0713	0.0718	0.0644	0.0819	0.0346	0.0343	0.0467
	500	0.0729	0.0737	0.0705	0.0838	0.0410	0.0426	0.0501
	1000	0.0775	0.0734	0.0796	0.0857	0.0431	0.0403	0.0540
0.3 0.5	100	0.0881	0.0828	0.0665	0.1021	0.0329	0.0303	0.0427
	200	0.0936	0.0906	0.0843	0.1101	0.0348	0.0368	0.0517
	300	0.0904	0.0901	0.0903	0.1096	0.0370	0.0397	0.0487
	500	0.0907	0.0900	0.0993	0.1141	0.0421	0.0396	0.0481
	1000	0.0928	0.0892	0.1076	0.1213	0.0447	0.0430	0.0544
0.5 0.5	100	0.1273	0.1200	0.1070	0.1535	0.0304	0.0310	0.0477
	200	0.1255	0.1199	0.1365	0.1705	0.0333	0.0333	0.0491
	300	0.1279	0.1252	0.1480	0.1797	0.0406	0.0393	0.0517
	500	0.1255	0.1190	0.1576	0.1815	0.0406	0.0381	0.0463
	1000	0.1292	0.1234	0.1785	0.1936	0.0445	0.0408	0.0520
0.5 0.8	100	0.1886	0.1792	0.1904	0.2557	0.0314	0.0310	0.0401
	200	0.1997	0.1927	0.2477	0.2940	0.0316	0.0373	0.0498
	300	0.1991	0.1887	0.2688	0.3131	0.0391	0.0370	0.0488
	500	0.2050	0.1957	0.3067	0.3405	0.0402	0.0380	0.0552
	1000	0.1980	0.1917	0.3229	0.3459	0.0436	0.0431	0.0482

The first and second columns represent different autoregressive coefficients and number of time points, respectively. Note that we used the residuals from the estimated AR(*p*) or ARMA(*p*,*q*) models by maximum likelihood estimate and the order selection was based on the Akaike Information criterion (AIC). The number of permutations was 1000. The pre-specified type I error was 0.05 and the number of replications was 10000

**Table 2:** The empirical type I error rates for the six different methods (the third to ninth column): PCC, SRCC, TLSA, permutation, LSAres (AR), LSAres (ARMA), and DDLSA, based on the ARMA(1,1) model

*ρ*_1_,*ρ*_2_	n	PCC	SRCC	TLSA	permutation	LSAres(AR)	LSAres(ARMA)	DDLSA
-0.5 -0.5	100	0.0524	0.0504	0.0314	0.0510	0.0303	0.0316	0.0325
	200	0.0506	0.0502	0.0357	0.0504	0.0359	0.0358	0.0369
	300	0.0469	0.0482	0.0343	0.0480	0.0399	0.0338	0.0346
	500	0.0487	0.0484	0.0390	0.0501	0.0396	0.0402	0.0399
	1000	0.0496	0.0491	0.0420	0.0495	0.0420	0.0414	0.0423
0 0	100	0.0835	0.0795	0.0620	0.0983	0.0297	0.0295	0.0400
	200	0.0830	0.0829	0.0784	0.1021	0.0372	0.0339	0.0443
	300	0.0878	0.0828	0.0853	0.1086	0.0406	0.0374	0.0428
	500	0.0823	0.0793	0.0890	0.1066	0.0411	0.0377	0.0433
	1000	0.0883	0.0847	0.1009	0.1130	0.0465	0.0445	0.0482
0.3 0.3	100	0.1401	0.1368	0.1356	0.1875	0.0300	0.0316	0.0399
	200	0.1350	0.1297	0.1539	0.1946	0.0376	0.0360	0.0407
	300	0.1380	0.1339	0.1732	0.2066	0.0361	0.0370	0.0432
	500	0.1377	0.1341	0.1839	0.2093	0.0376	0.0401	0.0442
	1000	0.1418	0.1368	0.1959	0.2141	0.0449	0.0435	0.0497
0.3 0.5	100	0.1659	0.1570	0.1583	0.2182	0.0285	0.0282	0.0372
	200	0.1662	0.1581	0.1942	0.2435	0.0368	0.0362	0.0401
	300	0.1663	0.1599	0.2220	0.2623	0.0401	0.0408	0.0438
	500	0.1616	0.1540	0.2339	0.2621	0.0415	0.0395	0.0444
	1000	0.1670	0.1611	0.2511	0.2742	0.0389	0.0415	0.0513
0.5 0.5	100	0.2012	0.1926	0.2126	0.2824	0.0326	0.0290	0.0390
	200	0.2016	0.1935	0.2668	0.3210	0.0377	0.0361	0.0416
	300	0.2012	0.1937	0.2827	0.3244	0.0394	0.0338	0.0415
	500	0.2118	0.2025	0.3188	0.3512	0.0376	0.0391	0.0481
	1000	0.2061	0.1966	0.3396	0.3651	0.0412	0.0447	0.0473
0.5 0.8	100	0.2620	0.2522	0.3050	0.3832	0.0297	0.0270	0.0329
	200	0.2737	0.2616	0.3842	0.4474	0.0328	0.0355	0.0370
	300	0.2624	0.2539	0.4056	0.4562	0.0394	0.0373	0.0425
	500	0.2577	0.2513	0.4439	0.4788	0.0438	0.0433	0.0433
	1000	0.2590	0.2492	0.4857	0.5136	0.0430	0.0415	0.0428

The first and second columns represent different autoregressive coefficients and number of time points, respectively. Note that we used the residuals from the estimated AR(*p*) or ARMA(*p*,*q*) models by maximum likelihood estimate and the order selection was based on the Akaike Information criterion (AIC). The number of permutations was 1000. The pre-specified type I error was 0.05 and the number of replications was 10000

**Table 3:** The empirical type I error rates for the six different methods (the third to ninth column): PCC, SRCC, TLSA, permutation, LSAres (AR), LSAres (ARMA), and DDLSA, based on the ARMA(1,1)-TAR(1) model

*ρ*_1_,*ρ*_2_	n	PCC	SRCC	TLSA	permutation	LSAres(AR)	LSAres(ARMA)	DDLSA
-0.5 -0.5	100	0.0490	0.0509	0.0295	0.0492	0.0273	0.0292	0.0309
	200	0.0511	0.0511	0.0369	0.0515	0.0341	0.0372	0.0387
	300	0.0495	0.0499	0.0393	0.0529	0.0383	0.0393	0.0400
	500	0.0511	0.0517	0.0414	0.0519	0.0388	0.0405	0.0407
	1000	0.0493	0.0508	0.0440	0.0496	0.0401	0.0419	0.0426
0 0	100	0.0494	0.0494	0.0294	0.0502	0.0283	0.0304	0.0329
	200	0.0532	0.0518	0.0330	0.0499	0.0323	0.0341	0.0359
	300	0.0487	0.0466	0.0368	0.0510	0.0368	0.0360	0.0377
	500	0.0776	0.0778	0.0841	0.0989	0.0373	0.0387	0.0445
	1000	0.0813	0.0813	0.0901	0.1005	0.0447	0.0400	0.0454
0.3 0.3	100	0.1172	0.1121	0.0955	0.1391	0.0280	0.0321	0.0431
	200	0.1181	0.1149	0.1191	0.1549	0.0329	0.0327	0.0438
	300	0.1181	0.1136	0.1277	0.1557	0.0349	0.0373	0.0460
	500	0.1135	0.1106	0.1436	0.1683	0.0411	0.0416	0.0469
	1000	0.1186	0.1122	0.1585	0.1748	0.0430	0.0460	0.0486
0.3 0.5	100	0.1245	0.1196	0.1098	0.1586	0.0336	0.0310	0.0396
	200	0.1369	0.1259	0.1400	0.1778	0.0315	0.0356	0.0449
	300	0.1350	0.1275	0.1545	0.1839	0.0421	0.0390	0.0454
	500	0.1355	0.1281	0.1690	0.1940	0.0423	0.0423	0.0466
	1000	0.1336	0.1339	0.1823	0.2014	0.0422	0.0407	0.0518
0.5 0.5	100	0.1584	0.1527	0.1527	0.2091	0.0280	0.0347	0.0423
	200	0.1589	0.1520	0.1827	0.2258	0.0352	0.0365	0.0433
	300	0.1604	0.1516	0.2004	0.2391	0.0382	0.0383	0.0429
	500	0.1545	0.1500	0.2203	0.2484	0.0358	0.0405	0.0472
	1000	0.1601	0.1507	0.2396	0.2609	0.0422	0.0425	0.0471
0.5 0.8	100	0.2160	0.2031	0.2282	0.2985	0.0312	0.0335	0.0401
	200	0.2157	0.2075	0.2858	0.3361	0.0351	0.0346	0.0399
	300	0.2194	0.2064	0.3158	0.3595	0.0391	0.0367	0.0431
	500	0.2144	0.2032	0.3221	0.3582	0.0402	0.0376	0.0444
	1000	0.2257	0.2083	0.3643	0.3920	0.0410	0.0423	0.0506

The first and second columns represent different autoregressive coefficients and number of time points, respectively. Note that we used the residuals from the estimated AR(*p*) or ARMA(*p*,*q*) models by maximum likelihood estimate and the order selection was based on the Akaike Information criterion (AIC). The number of permutations was 1000. The pre-specified type I error was 0.05 and the number of replications was 10000

Table 1 shows that, except for the case of *ρ*_1_=0,*ρ*_2_=0, the empirical type I error rates of PCC, SRCC, TLSA and the permutation approaches are all larger than the pre-specified type I error. When *ρ*_1_=0,*ρ*_2_=0, the empirical type I error rates of PCC, SRCC, TLSA and the permutation approaches are well controlled, which is reasonable as the time series are independent bivariate normally distributed. Further, the empirical type I error of TLSA is somewhat smaller than the significance level of 0.05 indicating that TLSA is conservative, consistent with findings in [12]. The results of LSAres and DDLSA are similar to that of TLSA. When *ρ*_1_≠0 and/or *ρ*_2_≠0, the PCC, SRCC, TLSA and the permutation approaches are not valid in the sense that their empirical type I error rates are much higher than the pre-specified type I error. On the other hand, both DDLSA and LSAres control the type I errors reasonably well under all the simulated scenarios. Their type I error approaches the significance level as the number of time points increases. The performances of LSAres(AR) and LSAres(ARMA) are similar.

Tables 2 and 3 show the similar results for ARMA(1,1) and ARMA(1,1)-TAR(1) models, respectively. Under the ARMA(1,1) and ARMA(1,1)-TAR(1) models with *ρ*_1_=−0.5,*ρ*_2_=−0.5, *X*_*t*_ are i.i.d.. Therefore, the type I error rates of PCC, SRCC, TLSA and permutation approaches are well controlled. However, the empirical type I error rates are much larger than the pre-specified type I error rate of 0.05 under all the other parameter settings. On the other hand, the type I error rates of LSAres and DDLSA are well controlled under all situations. Further, the type I error rates of both LSAres(AR) and LSAres(ARMA) are well controlled indicating that LSAres is applicable even when the generative model is mis-specified.

Finally, the simulation results for time delay *D*≠0 are presented in the Additional file 2: Table S1-S3.

### Comparing the power of LSAres and DDLSA

**Fig. 2:**
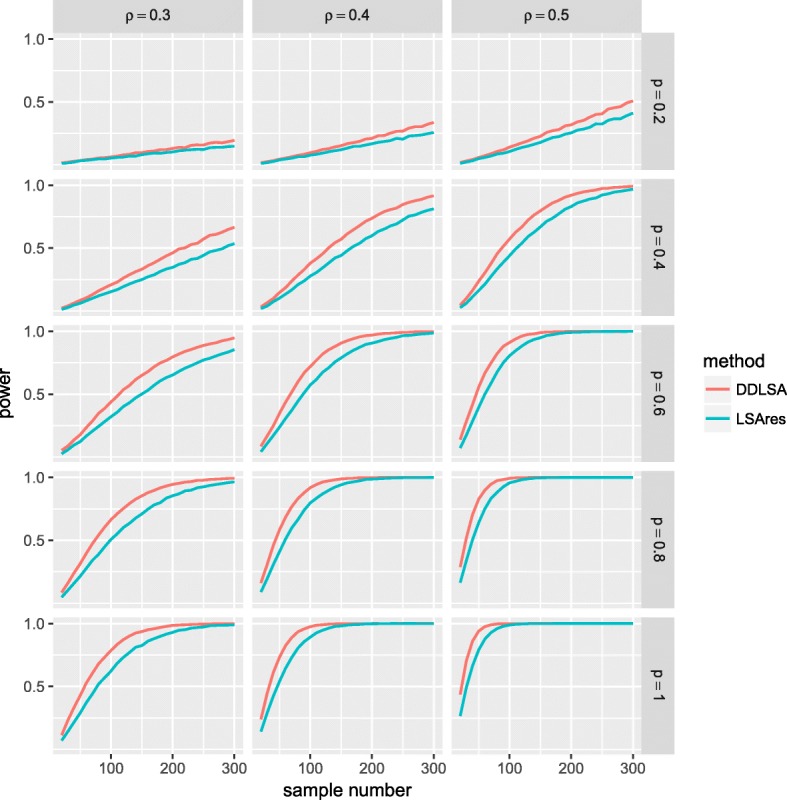
The power of LSAres and DDLSA in testing for the local association of two time series data under the local AR model. Ten thousand random samples were generated from the local AR model with *ρ*_1_=0.5. The LSAres approach used the residuals from the estimated ARMA(*p*,*q*) model by maximum likelihood estimate and the order was selected using the AIC criterion. The type I error is 0.05

Since PCC, SRCC, permutation and TLSA could not control type I error, we only investigated the power of LSAres and DDLSA. In the local AR model, we let *ρ*_1_=0.5, *ρ*=0.3,0.4,0.5, *p* from 0.2 to 1, and the number of time points *n* from 20 to 300. Figure 2 shows the power of DDLSA and LSAres as a function of the number of time points. The power of both LSAres and DDLSA increases with the number of time points *n*, percentage of correlation *p*, and serial correlation *ρ*. In particular, when the two time series are associated in 60% of the time interval (*p*=0.6) with correlation (*ρ*=0.5), the power of DDLSA is greater than 0.9 when the number of time points *n* is at least 100. Under the AR model, the power of DDLSA is higher than that of LSAres. Although we only show the results for *ρ*_1_=0.5 and time lag *D*=0, the results from other simulations with different autocorrelation parameters and time delays are similar to the result shown here. The simulation results under the local AR model with time delay *D*>0 are shown in Additional file 3: Fig. S1-S3.

**Fig. 3:**
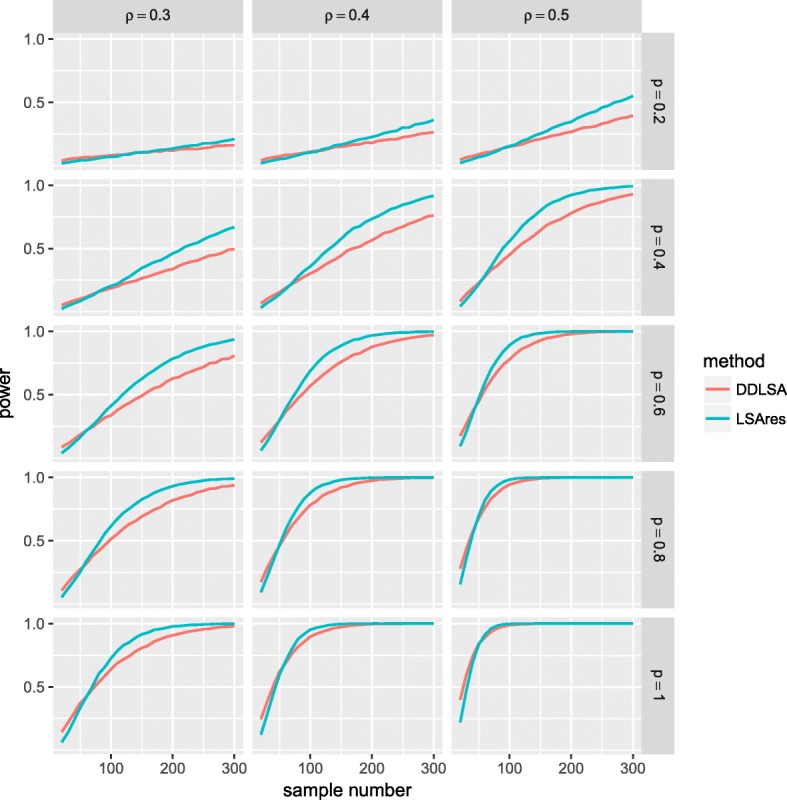
The power of LSAres and DDLSA in testing for the local association of two time series data under the bivariate AR model. Ten thousand random samples were generated from the bivariate AR model with *ρ*_1_=0.5,*ρ*_2_=0.5. The LSAres approach used the residuals from the estimated ARMA(*p*,*q*) model by maximum likelihood estimate and order was selected using the AIC criterion. The type I error is 0.05

Similar to the simulations under the local AR model, we also investigated the power of DDLSA and LSAres with different parameters under the bivariate AR model and the results are shown in Fig. 3. However, the power of LSAres is higher than that of DDLSA, different from the local AR model. Overall, LSAres in testing local association is more useful than DDLSA if we know that the time series come from the pre-defined model, such as the ARMA model. The simulated results for the power of DDLSA and LSAres under the bivariate AR(1) model with time delay *D*>0 are shown in Additional file 3: Fig. S4-S6.

### Significantly associated OTU pairs from the MPHM data set

**Fig. 4:**
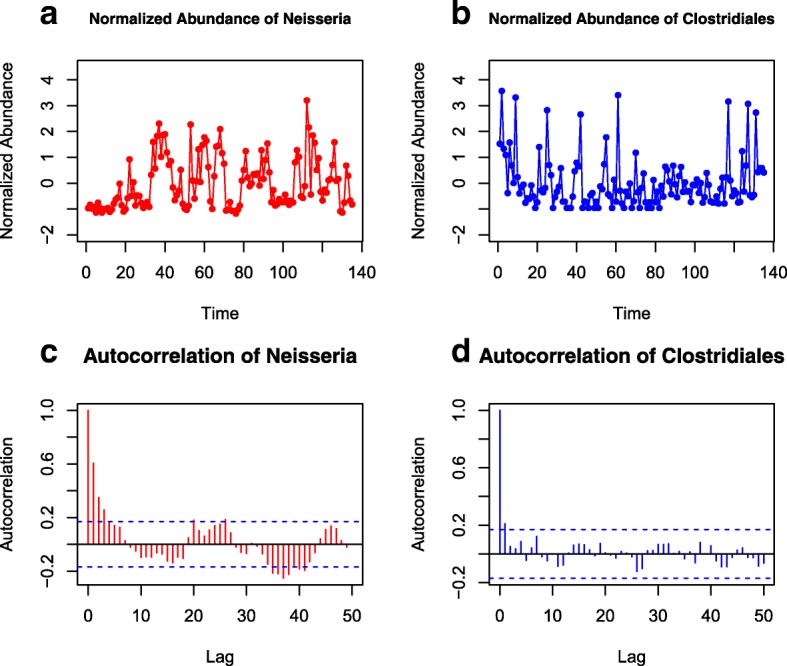
The standardized abundance of *Neisseria* (**a**) and *Clostridiales* (**b**) from the tongue time series of ‘F4’ in the MPHM dataset. The autocorrelograms (**c**, **d**) show the autocorrelation of the two time series responding to itself for different lags, respectively. The dashed line represents the critical value of the statistics $\pm \thinspace 1.96/\sqrt {n}$, where *n* is the number of time points of the time series. The region bounded by the dashed lines give the pointwise acceptance area for testing the null hypothesis that the autocorrelation functions of time series are zero at the 5% significance level

We analyzed the relationships among 41 OTUs that were observed in at least 60% of the tongue samples of individual ‘F4’. First, we found 21 significant autocorrelated OTUs among 41 OTUs using the Box-Ljung test [21] under the null hypothesis *H*_0_:*ρ*(*k*)=0 at the 5% significance level, where *ρ*(*k*) is the autocorrelation function for lag *k*. Figure 4 shows two autocorrelated OTUs. The first-order autocorrelation of *Neisseria* is 0.61 (*P*-value =1.96×10^−12^) indicating high autocorrelation. Although *Clostridiales* had relatively low autocorrelation (0.21), the hypothesis of no autocorrelation can still be rejected (*P*-value = 0.0148).

**Fig. 5:**
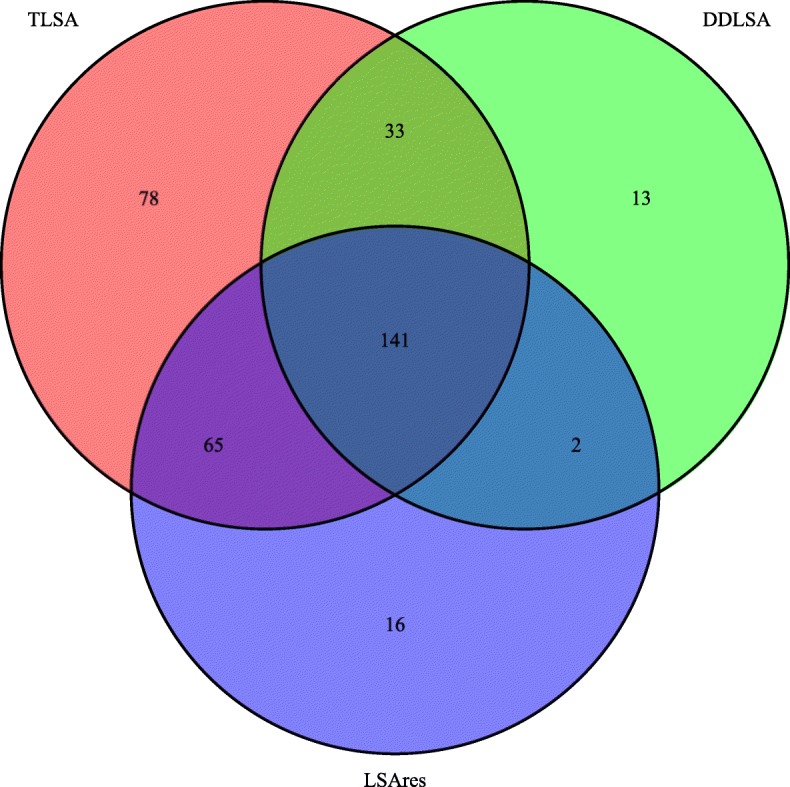
Venn diagram of the relationship among significant associated pairs found by the TLSA, DDLSA and LSAres in the MPHM dataset. Red, green and blue colors represent the number of pairs found by TLSA, DDLSA and LSAres, respectively

Second, we identified significantly locally associated OTU pairs with both *p*-value and false discovery rate (FDR) below 0.05 and compared the performance of TLSA, DDLSA and LSAres with time delay up to 3. For LSAres, the residuals were found based on the ARMA(p,q) model and the orders were selected based on the AIC criterion. In our study, we used FDR or Q-value to adjust for multiple hypothesis testing using the *qvalue* package in R [22]. Restricting the *p*-value *P*≤0.05 and q-value *Q*≤0.05, 317 pairs of significant associations are found among all 820 OTU pairs by TLSA, 189 by DDLSA, and 224 by LSAres, respectively (Table 4). Among the associations found by TLSA, 143 (∼ 45*%*) are not significant by DDLSA, and 111 (∼ 35*%*) are not significant by LSAres (Fig. 5). Such associations identified by TLSA but not by DDLSA or LSAres may be false positives caused by the autocorrelation of the raw data. If we combine associated pairs from DDLSA and LSAres, i.e. we define significant pairs as those found significant by either DDLSA or LSAres, 239 (∼ 89*%*) pairs out of 270 in total found by DDLSA or LSAres are also significant by TLSA. This finding is interesting, and it suggests that the combination of DDLSA and LSAres exhibits better performance than each alone. Note that DDLSA also finds some associations missed by LSAres and vice versa. For instance, DDLSA finds 189 and LSAres finds 224 significant associations but only 143 are found by both LSAres and DDLSA. Therefore, either DDLSA or LSAres is not a substitute but a complementary approach to the other one. For a comprehensive analysis of a data set, one should apply both approaches. Table 4 shows the results with more strict criteria of *P*≤0.01 and *Q*≤0.01.

**Table 4:** The numbers of significant associations found by TLSA, DDLSA and LSAres with different thresholds in the MPHM and PML data sets

		TLSA	DDLSA	LSAres	TLSA	DDLSA	LSAres
Dataset	# of OTUs	*P*≤0.01	*P*≤0.01	*P*≤0.01	*P*≤0.05	*P*≤0.05	*P*≤0.05
		*Q*≤0.01	*Q*≤0.01	*Q*≤0.01	*Q*≤0.05	*Q*≤0.05	*Q*≤0.05
MPHM F4 tongue	41	222	126	168	317	189	224
PML	75	413	227	36	761	371	98

**Fig. 6:**
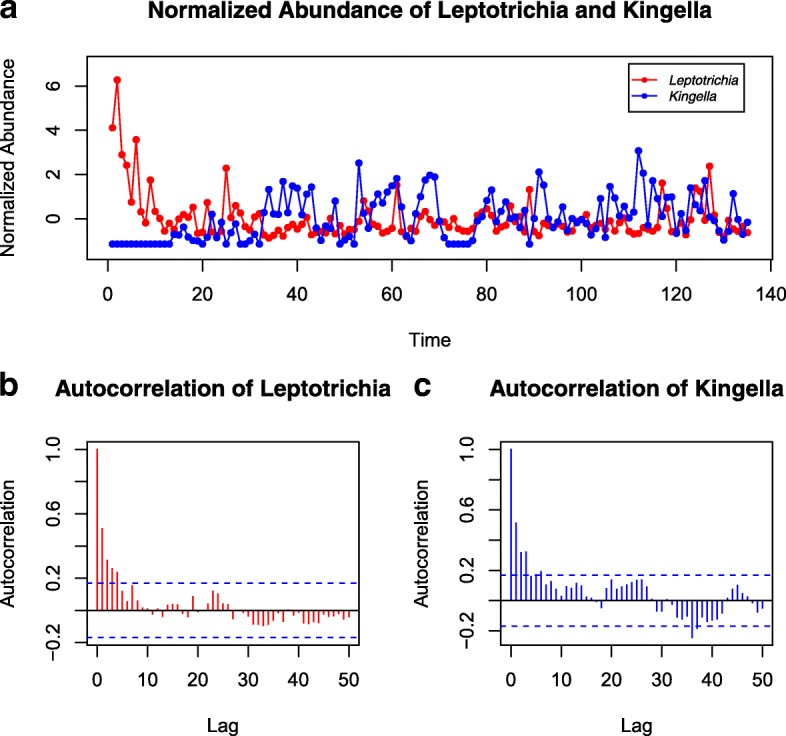
The standardized abundance of *Leptotrichia* and *Kingella* (**a**) from the tongue of ‘F4’ in the MPHM dataset. The autocorrelograms (**b**, **c**) of these bacterias show significant autocorrelation. The dashed line represents the critical value of the statistics $\pm \thinspace 1.96/\sqrt {n}$, where *n* is the number of time points of the time series. The region bounded by the dashed lines give the pointwise acceptance area for testing the null hypothesis that the autocorrelation functions of time series are zero at the 5% significance level

We carefully investigated one of the OTU pairs identified by TLSA but not by DDLSA and LSAres: *Leptotrichia* and *Kingella* (Fig. 6). The association is significant by TLSA within a time interval of length 129 starting from the first time point with 3 days delay where *Leptotrichia* precedes *Kingella* (*P*-value = 0.003 and Q-value = 0.007 by TLSA), while not significant by DDLSA (*P*-value = 0.16, Q-value = 0.38) and LSAres (*P*-value = 0.50, Q-value = 0.55). The autocorrelograms of the two OTUs show that both of them have the strong autocorrelation, where TLSA can’t control the type I error. However, DDLSA and LSAres work well in this situation.

In addition, we investigated if these site-specific significant associations are shared across the two individuals. Sørensen index *Q*_*s*_ [23] was used to evaluate the similarity between significant associations of the two samples from ‘F4’ and ‘M3’. We considered only the common OTUs in the two samples. The two individuals shared 40 and 41 OTUs in the feces and tongue samples, respectively. Let S1 and S2 be the sets of significant associations between common OTUs of the two samples. The Sorensen index is defined as $\frac {2|S_{1} \cap S_{2}|}{|S_{1}|+|S_{2}|}$, where *S*_1_∩*S*_2_ is the intersection of *S*_1_ and *S*_2_ and |·| is the number of OTU pairs in a set. Using LSAres, we identified 91 (*Q*_*s*_=0.35) and 177 (*Q*_*s*_=0.55) shared significant associations in the feces and tongue samples ‘F4’ and ‘M3’, respectively. Using DDLSA, the corresponding numbers are 61 (*Q*_*s*_=0.32) and 122 (*Q*_*s*_=0.46).

### Significantly associated OTU pairs from the PML data set

**Fig. 7:**
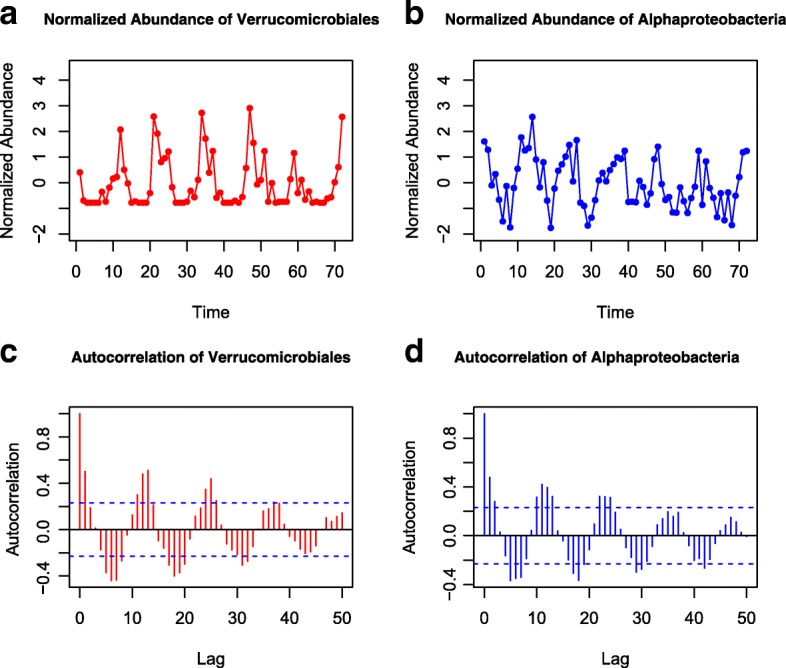
The standardized abundance of *Verrucomicrobia* (**a**) and *Alphaproteobacteria* (**b**) in the PML dataset. The autocorrelograms (**c**, **d**) show the autocorrelation of two time series responding to itself for different lags, respectively. Note that there are significant seasonal variations in the plot of OTUs and their autocorrelograms throughout the 6-year period

The seasonality of particular OTUs is obvious in their abundance profiles and autocorrelograms as shown in [20]. The stronger the seasonal periodicity, the more closely the autocorrelogram approaches a cyclical function. For example, there are significant seasonal cycles in the autocorrelograms of *Verrucomicrobiales* and *Alphaproteobacteria* (Fig. 7), and their periods are similar (about 1 year). Therefore, the abundance profiles of bacteria are possibly similar at the same time point of every year. However, the abundance may be somewhat different in some years. For example, both *Verrucomicrobiales* and *Alphaproteobacteria* are more abundant in the third year. In addition, a total of 33 out of 75 factors are significant autocorrelated based on the Box-Ljung test at the 5% significance level, including 9 environment factors and 24 OTUs. We applied TLSA, DDLSA and LSAres to obtain significant associations of these 75 factors and Table 4 shows the number of identified significant associations.

**Fig. 8:**
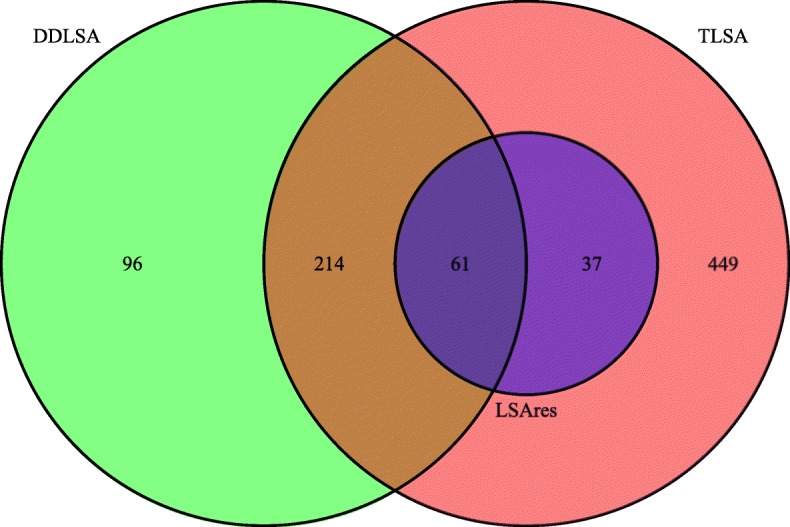
Venn diagram of the relationship among significant pairs found by the TLSA, DDLSA and LSAres in the PML dataset. Red, green and blue colors represent the number of pairs found by TLSA, DDLSA and LSAres, respectively

Among 2550 pairwise associations of all 75 factors, 761, 371 and 98 pairs were found significant with time delay 3 with both *P*-value and Q-value ≤ 0.05 by TLSA, DDLSA and LSAres, respectively. The relatively large number of significant associations identified by TLSA contain a large fraction of false positives since the dependency of the time series is not considered. The DDLSA and LSAres reduce the number of significant associations resulting from the factors’ autocorrelation. Figure 8 shows the Venn diagram illustrating the relationship of the sets of significant associations using the three approaches. There are 61 pairs found by all three methods. All the 98 associations found by LSAres are also significant by TLSA. This could be due to the periodicity of OTUs that makes ARMA model unsuitable for this dataset. We note that 486 (∼ 64*%*) out of 761 significant pairs by TLSA is non-significant by DDLSA, indicating that the autocorrelation of OTUs may lead to many false positives in the TLSA test. On the other hand, 275 out of the 371 (74%) significant associations found by DDLSA are also found by TLSA indicating high agreement with TLSA. If we combine the significant associations found by DDLSA and LSAres, 312 (∼ 76*%*) pairs out of 408 in total found by DDLSA or LSAres are also significant by TLSA. This result displays that the combination of DDLSA and LSAres exhibits better performance than each alone. In addition, the majority of associated OTU pairs found by TLSA and DDLSA are between the *Proteobacteria*, *Actinobacteria* and *Verrucomicrobia* phylum members, while those found by LSAres are between *Proteobacteria*, *Verrucomicrobia* and *Gemmatimonadetes* phylum members.

## Conclusions

The rapid development of high-throughput sequencing technology generates massive amounts of sequencing data effectively and economically. These developments make large scale human metagenomics studies in a wide range of environment possible. A variety of time series data from these studies brings great opportunities for statistical methods to gain insight into the temporal and spatial dynamics of biological systems. Therefore, for obtaining more accurate and efficient results, it’s necessary to consider the specific property of time series in these studies, such as autocorrelation.

In this paper, we developed a theoretical statistical significance approximation of local similarity score for dependent time series data, which substitutes long-run variance based on nonparametric kernel estimate for sample variance. Moreover, we developed another method to approximate the statistical significance by using raw data’s residuals from a predefined model. We considered different dependent time series models to evaluate the type I error and power of our methods compared with others, i.e. original TLSA, permutation test, PCC and SRCC. Results from our simulations showed that our methods can control type I error reasonably, but the other four approaches cannot. Through simulations, we showed that DDLSA performs better than LSAres for the local AR model, but LSAres works better than DDLSA in the bivariate AR model. Therefore, these two methods complement each other under different correlation scenarios. Using the MPHM and PML datasets, we demonstrated that DDLSA and LSAres reduced the redundant associations efficiently and captured the most possible relationships among OTUs in metagenomics studies of microbial communities. In addition, to obtain more complete sets of significant associations, we suggested to integrate the results from DDLSA and LSAres—apply DDLSA and LSAres to the data set simultaneously and combine the significant associations identified by at least one method as the final significant associations. This will reduce false negatives effectively.

However, one drawback of LSAres is the determination of the data generative model. If we presume data from a more complicated model, residuals from this model may seem like normally distributed but may lose too much information about the original data. We have to make a tradeoff between employing complicated models and preserving useful information. In the paper, we investigated the impact on type I error by considering AR and ARMA models as alternative models and both of them work well. In the future, we will continue to study the influence of model mis-specification.

We applied DDLSA and LSAres to time series data in microbial communities. In fact, they can be used in any type of data with the same length, such as medical (EEG or MEG signals), climate (temperature, solar irradiance, river runoff or rainfall) and economic (stock price) time series data. The time-delay associations of EEG time series play an important role in discovering new information about the activity of brain [24]. Climate time series often exhibit positive serial dependence [18]. Potentially local and time delayed associations are widespread in climate data, but it will increase the number of false positives if we use TLSA to calculate the statistical significance of their LS scores, while DDLSA and LSAres can overcame this problem.

## Additional files

Additional file 1 Appendix. Theoretical approximation of LSA statistical significance for i.i.d. or Markov time series and derivation of the asymptotic distribution of the LS score statistics. (PDF 259 kb)

Additional file 2 **Table S1-S3.** Type I errors of TLSA, LSAres and DDLSA tests under the AR(1), ARMA(1,1) and ARMA(1,1)-TAR(1) models with time delay *D*≠0, respectively. (PDF 551 kb)

Additional file 3 **Figure S1-S6.** Power of LSAres and DDLSA for local AR model and bivariate AR model with different time delays(D). (PDF 1115 kb)

## Abbreviations

AR: Autoregressive model
ARMA: Autoregressive moving average model
DDLSA: Data-driven local similarity analysis
EEG: Electroencephalogram
FDR: False discovery rate
i.i.d.: independent identical distributed
LS: Local similarity
LSA: Local similarity analysis
LSAres: Local similarity analysis based on residuals
MEG: Magnetoencephalogram
MPHM: Moving pictures of the human microbiome (Dataset)
OTU: Operational taxonomic units
PCC: Pearson’s correlation coefficient
PML: Polymouth marine laboratory (Dataset)
SRCC: Spearman’s rank correlation coefficient
TAR: Threshold autoregressive model
TLSA: Theoretical significance of local similarity analysis
