# Supplementary material for: Statistical significance approximation for local similarity analysis of dependent time series data
Source: BMC Bioinformatics. 2019 Jan 28;20:53. doi: 10.1186/s12859-019-2595-x (PMC6348690; doi:10.1186/s12859-019-2595-x)
Supplement: Supplementary file 1 — Appendix. Theoretical approximation of LSA statistical significance for i.i.d. or Markov time series and derivation of the asymptotic distribution of the LS score statistics. (PDF 259 kb) [file 12859_2019_2595_MOESM1_ESM.pdf]

Statistical significance approximation for local similarity analysis of dependent time series data: Supplementary Information

Fang Zhang[1],Fengzhu Sun[2,3], and Yihui Luan[1*]

[1] School of Mathematics, Shandong University, Jinan, Shandong, 250100, China

[2] Quantitative and Computational Biology Program, Department of Biological Sciences, University of Southern California, 1050 Childs Way, Los Angeles, CA 90089, USA

[3] Institute of Science and Technology for Brain-inspired Intelligence, Fudan University, Shanghai, 200433, China

1 Original local similarity analysis

Consider two time series X_t and $Y_t, t = 1, \cdots, n$ with mean 0. The local similarity analysis [1, 2] was used to find intervals of the same length in two time series, i.e. $I = [i, i + k - 1]$ and $J = [j, j + k - 1]$, $|i - j| \leq D$ such that the absolute value of $S_{i,j,k} = \sum_{l=0}^{k-1} X_{i+l}Y_{j+l}$ is maximized, which is referred to as local similarity (LS) score with maximum predefined time delay D:

$$LS(D) = \max_{0 \leq i,j,k \leq n; |i-j| \leq D} \left| \sum_{l=0}^{k-1} X_{i+l}Y_{j+l} \right|. \tag{1}$$

The parameter D is chosen by the investigator to reflect the maximum time delays which the investigator is interested in.

Given X_t, Y_t and their LS score, we want to test if the two sequences are associated. We regard this as a hypothesis testing problem. The null hypothesis H_0 is that the two time series X_t and Y_t are not associated, and LS score is the statistic. In earlier studies [1, 2], permutation test was used to obtain the statistical significance (p-value) of the LS score, namely the probability that the LS score would be the same or greater than the actual observed value under the null hypothesis H_0. Particularly, one of the time series data were fixed and the other one were permutated many times, and the resulting LS score was obtained using the dynamic programming algorithm. The p-value was approximated by the fraction of times the LS score of the permuted data is larger than the LS score of the actual data.

2 Theoretical approximation of statistical significance of LS score for i.i.d. or Markov time series

Using the theory of the Bachelier-Wiener processes, Feller [3] investigated the asymptotic distribution of the range of the partial sum of i.i.d. random variables. Daudin et al. [4] studied the asymptotic behavior of the local score of either i.i.d. random variables with finite second moment or a stationary and irreducible Markov chain taken values on a finite subset of $\mathbb{R}$. Based on these results, Xia et al. [5]

*Corresponding author. Email: yhluan@sdu.edu.cn

approximated the statistical significance of the LS score for i.i.d. or Markovian time series. Specifically, given X_t and Y_t, let $Z_t = X_tY_t$. If X_t and Y_t are i.i.d. random variables, Z_t are also i.i.d. under the null hypothesis H_0. Let $E(Z_t) = 0$ and $var(Z_t) = \sigma^2$ if X_t and Y_t are i.i.d.. For Markov time series, let ϕ be the stationary distribution of Z_t, $E(Z_t) = 0$, and $\sigma^2 = E_\phi(Z_1^2) + 2\sum_{k=1}^{\infty} E_\phi(Z_1 Z_{k+1})$. Then the statistical significance of LS score of X_t and Y_t with maximum time delay D was approximated by

$$P(LS(D) \geq s_D) = P\left(\frac{LS(D)}{\sigma\sqrt{n}} \geq \frac{s_D}{\sigma\sqrt{n}}\right) = \mathcal{L}_D\left(\frac{s_D}{\sigma\sqrt{n}}\right), \tag{2}$$

where s_D is the LS score of X_t and Y_t, and the tail probability distribution function $\mathcal{L}_D(x)$ is defined as

$$\mathcal{L}_D(x) \approx 1 - 8^{2D+1}\left[\sum_{k=1}^{\infty}\left\{\frac{1}{x^2} + \frac{1}{(2k-1)^2\pi^2}\right\}\exp\left\{-\frac{(2k-1)^2\pi^2}{2x^2}\right\}\right]^{2D+1}. \tag{3}$$

As the summation in Eq. (3) increases monotonously and converges to a certain value as n tends to infinity, we can use a small tolerance ϵ to decide when to stop the summation in practical calculation, such as 0.00001. Specifically, the summation will be stopped when the absolute value of the difference between k-th and $(k+1)$-th partial summation is less than ϵ. Another option is that we only calculate the first K items of the infinite sum for a given K, such as 200.

3 Derivation of the asymptotic distribution of the LS score for dependent time series data

Assume X_t and Y_t are weakly stationary time series with mean 0. Under the null hypothesis H_0, $E(Z_t) = 0$. Let $S_k = \sum_{i=1}^{k} Z_i, k = 1, 2, \cdots, n$, $M_n = \max\{0, S_1, \cdots, S_n\}$ and $m_n = \min\{0, S_1, S_2, \cdots, S_n\}$. Define the range $R_n = M_n - m_n$. We can see that $R_n = LS(0)$. Moreover, let the standardized partial sum process

$$B_n(t) = \frac{1}{\omega\sqrt{n}} S_{[nt]}, \quad 0 \leq t \leq 1. \tag{4}$$

where ω is a scale parameter, $\omega^2 = \lim_{n\to\infty} \frac{E(S_n^2)}{n}$ and $[x]$ denotes the integer part of x. Several investigators showed that under appropriate regularity conditions the weak convergence

$$B_n(t) \Rightarrow W(t) \quad (n \to \infty) \tag{5}$$

holds [6–8]. Here $(W(t) : t \in [0, 1])$ is the standard Brownian motion.

If the generative model of the real data is known, the long-run variance can be calculated by the limit of $Var(S_n/\sqrt{n})$ in a closed form. For example, suppose that Z_t come from the AR(1) model such that $Z_t = \phi Z_{t-1} + \varepsilon_t$, where ε_t are standard normal random variables. The long-run variance of Z_t is computed as $\omega_Z^2 = 1/(1-\phi)^2$, where ϕ should be estimated by some methods, such as least square methods or maximum likelihood estimate. However, in reality we usually do not know the precise data-generating model. In such situations, the nonparametric estimator can be used to estimate ω, which have been shown in the main text.

Let

$$H_n = \max_{1\leq i\leq j\leq n}(S_j - S_i) = \max_{1\leq i\leq j\leq n}(Z_{i+1} + \cdots + Z_j) \tag{6}$$

be the maximum subinterval sum assigned to $\{Z_i\}_{i=1}^n$. Following Theorem 1 and Proposition 2 in [4], the limit distribution of H_n is given in the next theorem.

Theorem 1. *Let $\{Z_i\}_{i=1}^n$ be a stationary sequence with zero expectation and finite second moment. Let $\mathcal{F}_k$ be a σ-field generated by Z_i with indices $i \leq k$. Assume that $\sum_{n=1}^{\infty} \frac{\|E(S_n|\mathcal{F}_0)\|}{n^{3/2}} < \infty$, where $\|X\| = \sqrt{E(X^2)}$. Then $H_n/(\omega\sqrt{n})$ converges in distribution to W^*, where ω is the long-run variance of Z_n, $W^* = \max_{0\leq v\leq 1}|W(v)|$ and $W(t)$ is the standard Brownian motion. Moreover, if $\hat{\omega}_n$ is a weakly consistent estimator of ω, $H_n/(\hat{\omega}_n\sqrt{n})$ converges in distribution to W^*.*

Proof. *Let* $(S_n)_{n\geq 0}$ *be the random walk:*

$$S_0 = 0, S_n = \sum_{k=1}^{n} Z_k, n \geq 1$$

As $\{Z_i\}_{i=1}^n$ *be a stationary sequence with zero expectation and finite second moment, given an integer* $N \geq 0$*, we consider the piecewise linear process* $B^{(N)}(t)$

$$\begin{cases} B^{(N)}(\frac{k}{N}) = \frac{1}{\omega\sqrt{N}}(S_k - E(S_k)) = \frac{1}{\omega\sqrt{N}} S_k, k \geq 0, \\ t \mapsto B^{(N)}(t) \text{ is linear on each interval of the form } [\frac{k}{N}, \frac{k+1}{N}]. \end{cases} \tag{7}$$

Recall that

$$H_k = \max_{1\leq i\leq j\leq k}(S_j - S_i), k \geq 0.$$

Let us introduce the linear interpolation of $(H_k)_{k\geq 0}$*. This function* $(H^{(N)}(t); t \geq 0)$ *depending on the parameter* N *is defined as follows:*

$$H^{(N)}(t) = \frac{1}{\sqrt{N}} \left\{ H_{[Nt]} + (Nt - [Nt])(H_{[Nt]+1} - H_{[Nt]}) \right\}, t \geq 0. \tag{8}$$

Relation (7) implies

$$S_k = \omega\sqrt{N} B^{(N)}\left(\frac{k}{N}\right). \tag{9}$$

Then

$$\begin{aligned} H_{[Nt]} &= \omega\sqrt{N} \max_{0\leq i\leq j\leq [Nt]} \left\{ B^{(N)}\left(\frac{j}{N}\right) - B^{(N)}\left(\frac{i}{N}\right) \right\} \\ &= \omega\sqrt{N} \max_{0\leq i/N\leq j/N\leq [Nt]/N} \left\{ B^{(N)}\left(\frac{j}{N}\right) - B^{(N)}\left(\frac{i}{N}\right) \right\}. \end{aligned}$$

$B^{(N)}$ *is piecewise linear, then the maximum on* $\{0 \leq i/N \leq j/N \leq [Nt]/N\}$ *is equal to the maximum on* $0 \leq u \leq v \leq [Nt]/N$ *and*

$$H_{[Nt]} = \omega\sqrt{N} \max_{0\leq u\leq v\leq [Nt]/N} \left\{ B^{(N)}(v)) - B^{(N)}(u) \right\}$$

Finally $H^{(N)}(t)$ *can be written as follows:*

$$H^{(N)}(t) = \omega \left(\max_{0\leq u\leq v\leq [Nt]/N} \left\{ B^{(N)}(v) - B^{(N)}(u) \right\} + R_N(t) \right), \tag{10}$$

$$\begin{aligned} R_N(t) =& (Nt - [Nt]) \left(\max_{0\leq u\leq v\leq ([Nt]+1)/N} \left\{ B^{(N)}(v) - B^{(N)}(u) \right\} \right. \\ & \left. - \max_{0\leq u\leq v\leq [Nt]/N} \left\{ B^{(N)}(v) - B^{(N)}(u) \right\} \right). \end{aligned} \tag{11}$$

Since the process $B_n(t)$ *(4) converges to a standard Brownian motion* $W(t)$*,* $B^{(N)}$ *also converges to the standard Brownian motion* $W(t)$*. For any* $t \in [0, 1]$*,*

$$\max_{0\leq u\leq v\leq ([Nt]+1)/N} \left\{ B^{(N)}(v) - B^{(N)}(u) \right\} \xrightarrow[N\to\infty]{a.s.} \max_{0\leq u\leq v\leq t} \{W(v) - W(u)\},$$

$$\max_{0\leq u\leq v\leq [Nt]/N} \left\{ B^{(N)}(v) - B^{(N)}(u) \right\} \xrightarrow[N\to\infty]{a.s.} \max_{0\leq u\leq v\leq t} \{W(v) - W(u)\}$$

Moreover, as $0 \leq Nt - [Nt] \leq 1$*, then*

$$R_N(t) \xrightarrow[N\to\infty]{a.s.} 0 \quad \text{uniformly in } t \in [0, 1].$$

Hence

$$(H^{(N)}(t), 0 \le t \le 1) \xrightarrow[N\to\infty]{a.s.} \left(\omega \max_{0\le u\le v\le t} \{W(v) - W(u)\}\,; 0 \le t \le 1\right). \tag{12}$$

We denote $\xi(t) = \max_{0\le u\le v\le t}\{W(v) - W(u)\} = \max_{0\le v\le t}\{W(v) - I(v)\}$ *where* $I(v) = \min_{0\le u\le v} W(u)$. *Recall that Paul Lévy's theorem ([9], Chapter II, Theorem 2.3) gives us the following identity:*

$$(W(v) - I(v), v \ge 0) \stackrel{(d)}{=} (|W_v|, v \ge 0)$$

where $\stackrel{(d)}{=}$ *signifies equality in distribution. Therefore,*

$$\frac{H_n}{\omega\sqrt{n}} = \frac{1}{\omega} H^{(n)}\left(\frac{n}{n}\right) = \frac{1}{\omega} H^{(n)}(1) \xrightarrow[n\to\infty]{a.s.} \max_{0\le v\le 1} |W(v)|.$$

Moreover, if $\hat{\omega}_n$ *is a weakly consistent estimator of* ω. *Then*

$$\hat{\omega}_n \xrightarrow[n\to\infty]{d} \omega,$$

where $\stackrel{d}{\to}$ *denotes convergence in distribution. From Slutsky's theorem, we obtain*

$$\frac{H_n}{\hat{\omega}_n\sqrt{n}} \xrightarrow[n\to\infty]{d} \max_{0\le v\le 1} |W(v)|.$$

□

Proposition 1. *Under the same conditions as Theorem 1,*

$$\lim_{n\to\infty} P\left(\frac{H_n}{\omega\sqrt{n}} \le x\right) = \frac{4}{\pi}\sum_{k=0}^{\infty} \frac{(-1)^k}{2k+1} \exp\left\{-\frac{(2k+1)^2\pi^2}{8x^2}\right\}. \tag{13}$$

This formula can be found in [4].

Define $L_n = -\min_{0\le i<j\le n}(S_j - S_i)$, then $R_n = \max(H_n, L_n)$. Here we assume the distribution of $\{Z_i\}_{i=1}^n$ is symmetric. since $E(Z_t) = 0$, L_n has the same limiting distribution as H_n. When x is large, the probability of $\{H_n \ge x\}\bigcap\{L_n \ge x\}$ will be small and the distribution function of $R_n/(\omega\sqrt{n})$ can be approximated by

$$\begin{aligned} P\left(\frac{R_n}{\omega\sqrt{n}} \ge x\right) &= P\left(\frac{\max(H_n, L_n)}{\omega\sqrt{n}} \ge x\right) \\ &= P\left(\left\{\frac{H_n}{\omega\sqrt{n}} \ge x\right\}\bigcup\left\{\frac{L_n}{\omega\sqrt{n}} \ge x\right\}\right) \\ &\approx P\left(\frac{H_n}{\omega\sqrt{n}} \ge x\right) + P\left(\frac{L_n}{\omega\sqrt{n}} \ge x\right) \\ &= 2P\left(\frac{H_n}{\omega\sqrt{n}} \ge x\right) \\ &= 2\left[1 - \frac{4}{\pi}\sum_{k=0}^{\infty}\frac{(-1)^k}{2k+1}\exp\left\{-\frac{(2k+1)^2\pi^2}{8x^2}\right\}\right]. \end{aligned} \tag{14}$$

The approximation is close to the true p-value when x is large enough. In addition, it is similar to Eq. (3) when $x \ge 2$. Thus, we apply Eq. (3) to calculate the approximate statistical significance of local similarity score only by replacing σ with ω, i.e.

$$\lim_{n\to\infty} P\left(\frac{R_n}{\omega\sqrt{n}} \ge x\right) = 1 - 8\sum_{k=1}^{\infty}\left\{\frac{1}{x^2} + \frac{1}{(2k-1)^2\pi^2}\right\}\exp\left\{-\frac{(2k-1)^2\pi^2}{2x^2}\right\}. \tag{15}$$

Following an analogical inference as in [5], under the null hypothesis that two time series X_t and Y_t are not related, we let $S_n^{(d)}$ be the local similarity score with no time delay for the pair of series $X_1, X_2, \cdots, X_n$ and $Y_{1+d}, Y_{2+d}, \cdots, Y_{n+d}$ ($d = 0, \pm 1, \pm 2, \cdots, \pm D, D > 0$). When the subscript is outside the range $[1, n]$, the corresponding outside data is not considered when the LS score is calculated. Let $Z_t^{(d)} = X_t Y_{t+d}, t = 1, 2, \cdots, n$, then $S_n^{(d)} = R_n(Z_t^{(d)})$. When n is sufficiently large, $S_n^{(d)}$ can be regarded

as approximately identical distributed. The distribution function of $S_n^{(d)}/(\omega\sqrt{n})$ can be approximate by Eq. (15). Note that $LS(D) = \max_{d=-D}^{D} S_n(d)$. To infer an approximate distribution function of $LS(D)$, we assume that $S_n^{(d)}, d = 0, \pm 1, \pm 2, \cdots, \pm D$ are independent although they are not. The statistical significance of LS score of X_t and Y_t with time delay up to D is approximately equal to

$$\begin{aligned}
\mathcal{L}_D(x) &= P\left(\frac{LS(D)}{\omega\sqrt{n}} \geq x\right) \\
&= 1 - P\left(\frac{LS(D)}{\omega\sqrt{n}} \leq x\right) \\
&\approx 1 - \prod_{d=-D}^{D} P\left(\frac{S_n^{(d)}}{\omega\sqrt{n}} \leq x\right) \\
&\approx 1 - 8^{2D+1}\left[\sum_{k=1}^{\infty}\left\{\frac{1}{x^2} + \frac{1}{(2k-1)^2\pi^2}\right\}\exp\left\{-\frac{(2k-1)^2\pi^2}{2x^2}\right\}\right]^{2D+1}.
\end{aligned} \tag{16}$$

References

[1] Qian J, Dolled-Filhart M, Lin J, Yu H, Gerstein M. Beyond synexpression relationships: local clustering of time-shifted and inverted gene expression profiles identifies new, biologically relevant interactions. J Mol Biol. 2001;314(5):1053-1066.

[2] Ruan Q, Dutta D, Schwalbach MS, Steele JA, Fuhrman JA, Sun F. Local similarity analysis reveals unique associations among marine bacterioplankton species and environmental factors. Bioinforma. 2006;22(20):2532-2538.

[3] Feller W. The asymptotic distribution of the range of sums of independent random variables. Ann Math Stat. 1951;22(3):427-432.

[4] Daudin JJ, Etienne MP, Vallois P. Asymptotic behavior of the local score of independent and identically distributed random sequences. Stoch Proc Appl. 2003;107(1):1-28.

[5] Xia LC, Ai DM, Cram J, Fuhrman JA, Sun FZ. Efficient statistical significance approximation for local similarity analysis of high-throughput time series data. Bioinforma. 2013;29(2):230-723.

[6] Bradley RC. Introduction to Strong Mixing Conditions. Heber City: Kendrick Press; 2007.

[7] Dedecker J, Doukhan P, Lang G, Leon JR, Louhichi S, Prieur C. Weak Dependence: With Examples and Applications. Lecture Notes in Statistics 190. New York: Springer; 2007.

[8] Peligrad M, Utev S. A new maximal inequality and invariance principle for stationary sequences. Ann Probab. 2005;33(2):798-815.

[9] Revuz D, Yor M. Continuous Martingales and Brownian Motion. Berlin:Springer; 1991.
