# Supplementary material for: Statistical significance approximation for local similarity analysis of dependent time series data
Source: BMC Bioinformatics. 2019 Jan 28;20:53. doi: 10.1186/s12859-019-2595-x (PMC6348690; doi:10.1186/s12859-019-2595-x)
Supplement: Supplementary file 2 — Table S1-S3. Type I errors of TLSA, LSAres and DDLSA tests under the AR(1), ARMA(1,1) and ARMA(1,1)-TAR(1) models with time delay D≠0, respectively. (PDF 551 kb) [file 12859_2019_2595_MOESM2_ESM.pdf]

Table S1: The empirical type I error rates for different methods: TLSA, LSAres(AR), LSAres(ARMA) and DDLSA, based on the AR(1) model with different time delays (D=1, 2, 3). The first and second columns represent different autoregressive coefficients and number of time points, respectively. Note that we used the residuals from the estimated AR(*p*) or ARMA(*p*,*q*) models by maximum likelihood estimate and the order selection was based on the Akaike Information criterion (AIC). The number of permutations was 1000. The pre-specified type I error was 0.05 and the number of replications was 10000.

		D=1				D=2				D=3			
ρ_1, ρ_2	n	TLSA	LSAres (AR)	LSAres(ARMA)	DDLSA	TLSA	LSAres (AR)	LSAres(ARMA)	DDLSA	TLSA	LSAres(AR)	LSAres(ARMA)	DDLSA
−0.5 − 0.5	100	0.1087	0.0280	0.0258	0.0329	0.1124	0.0225	0.0249	0.0314	0.1131	0.0207	0.0207	0.0305
	200	0.1404	0.0324	0.0318	0.0350	0.1482	0.0304	0.0338	0.0366	0.1490	0.0308	0.0293	0.0366
	300	0.1458	0.0340	0.0363	0.0354	0.1622	0.0329	0.0346	0.0347	0.1631	0.0343	0.0315	0.0343
	500	0.1584	0.0382	0.0400	0.0370	0.1727	0.0359	0.0374	0.0353	0.1851	0.0354	0.0373	0.0365
	1000	0.1735	0.0392	0.0439	0.0428	0.1869	0.0368	0.0392	0.0412	0.2023	0.0433	0.0426	0.0401
0 0	100	0.0266	0.0247	0.0264	0.0264	0.0225	0.0224	0.0213	0.0237	0.0206	0.0212	0.0245	0.0223
	200	0.0323	0.0323	0.0324	0.0338	0.0307	0.0299	0.0322	0.0320	0.0304	0.0302	0.0302	0.0302
	300	0.0343	0.0363	0.0370	0.0350	0.0348	0.0343	0.0348	0.0334	0.0335	0.0371	0.0337	0.0336
	500	0.0396	0.0420	0.0407	0.0410	0.0379	0.0389	0.0367	0.0376	0.0374	0.0377	0.0391	0.0371
	1000	0.0407	0.0402	0.0405	0.0406	0.0390	0.0430	0.0406	0.0386	0.0416	0.0411	0.0422	0.0406
0.3 0.3	100	0.0480	0.0225	0.0264	0.0382	0.0462	0.0229	0.0239	0.0363	0.0440	0.0204	0.0209	0.0345
	200	0.0550	0.0340	0.0349	0.0402	0.0575	0.0327	0.0297	0.0403	0.0610	0.0281	0.0309	0.0421
	300	0.0658	0.0377	0.0332	0.0480	0.0667	0.0348	0.0379	0.0453	0.0698	0.0346	0.0322	0.0442
	500	0.0742	0.0387	0.0372	0.0483	0.0751	0.0374	0.0396	0.0476	0.0767	0.0392	0.0379	0.0481
	1000	0.0811	0.0423	0.0424	0.0481	0.0847	0.0428	0.0395	0.0473	0.0844	0.0415	0.0392	0.0479
0.3 0.5	100	0.0629	0.0257	0.0282	0.0365	0.0617	0.0234	0.0231	0.0346	0.0623	0.0222	0.0191	0.0344
	200	0.0835	0.0322	0.0336	0.0446	0.0872	0.0273	0.0313	0.0435	0.0938	0.0300	0.0287	0.0417
	300	0.0921	0.0353	0.0353	0.0441	0.0964	0.0360	0.0289	0.0424	0.1011	0.0332	0.0338	0.0422
	500	0.0973	0.0417	0.0423	0.0396	0.1063	0.0384	0.0367	0.0454	0.1092	0.0366	0.0363	0.0443
	1000	0.1102	0.0396	0.0400	0.0457	0.1144	0.0392	0.0411	0.0449	0.1229	0.0384	0.0424	0.0423
0.5 0.5	100	0.1055	0.0264	0.0262	0.0397	0.1106	0.0223	0.0214	0.0371	0.1129	0.0198	0.0218	0.0369
	200	0.1338	0.0320	0.0350	0.0399	0.1440	0.0278	0.0311	0.0381	0.1513	0.0303	0.0315	0.0340
	300	0.1505	0.0402	0.0331	0.0428	0.1650	0.0337	0.0347	0.0408	0.1743	0.0331	0.0338	0.0399
	500	0.1561	0.0378	0.0368	0.0396	0.1676	0.0368	0.0382	0.0373	0.1806	0.0361	0.0361	0.0365
	1000	0.1734	0.0409	0.0453	0.0411	0.1853	0.0417	0.0394	0.0375	0.2028	0.0392	0.0395	0.0395
0.5 0.8	100	0.1709	0.0246	0.0231	0.0250	0.1759	0.0238	0.0235	0.0231	0.1832	0.0194	0.0212	0.0211
	200	0.2266	0.0333	0.0325	0.0309	0.2370	0.0297	0.0325	0.0289	0.2535	0.0309	0.0308	0.0264
	300	0.2443	0.0400	0.0372	0.0304	0.2508	0.0334	0.0331	0.0284	0.2641	0.0338	0.0347	0.0245
	500	0.2766	0.0383	0.0382	0.0350	0.2850	0.0414	0.0364	0.0301	0.2990	0.0383	0.0354	0.0307
	1000	0.2919	0.0434	0.0420	0.0361	0.3077	0.0411	0.0389	0.0315	0.3246	0.0424	0.0417	0.0272

Table S2: The empirical type I error rates for different methods: TLSA, LSAres(AR), LSAres(ARMA) and DDLSA, based on the ARMA(1,1) model with different time delays (D=1, 2, 3). The first and second columns represent different autoregressive coefficients and number of time points, respectively. Note that we used the residuals from the estimated AR(*p*) or ARMA(*p*,*q*) models by maximum likelihood estimate and the order selection was based on the Akaike Information criterion (AIC). The number of permutations was 1000. The pre-specified type I error was 0.05 and the number of replications was 10000.

		D=1				D=2				D=3			
ρ_1, ρ_2	n	TLSA	LSAres (AR)	LSAres(ARMA)	DDLSA	TLSA	LSAres (AR)	LSAres(ARMA)	DDLSA	TLSA	LSAres(AR)	LSAres(ARMA)	DDLSA
−0.5 − 0.5	100	0.0265	0.0278	0.0269	0.0285	0.0260	0.0276	0.0261	0.0283	0.0240	0.0214	0.0239	0.0263
	200	0.0327	0.0323	0.0331	0.0359	0.0322	0.0353	0.0324	0.0327	0.0300	0.0294	0.0293	0.0326
	300	0.0356	0.0320	0.0347	0.0367	0.0344	0.0309	0.0333	0.0347	0.0313	0.0324	0.0313	0.0333
	500	0.0382	0.0386	0.0388	0.0399	0.0369	0.0381	0.0371	0.0379	0.0395	0.0389	0.0392	0.0402
	1000	0.0416	0.0426	0.0422	0.0413	0.0426	0.0411	0.0423	0.0428	0.0444	0.0409	0.0433	0.0456
0 0	100	0.0621	0.0262	0.0240	0.0373	0.0654	0.0274	0.0241	0.0388	0.0656	0.0245	0.0213	0.0387
	200	0.0778	0.0303	0.0323	0.0385	0.0842	0.0303	0.0273	0.0394	0.0874	0.0314	0.0281	0.0397
	300	0.0919	0.0322	0.0361	0.0433	0.0921	0.0356	0.0333	0.0413	0.1003	0.0374	0.0345	0.0407
	500	0.0921	0.0420	0.0370	0.0389	0.0997	0.0394	0.0338	0.0388	0.1033	0.0400	0.0348	0.0409
	1000	0.1060	0.0427	0.0440	0.0424	0.1156	0.0408	0.0436	0.0398	0.1170	0.0383	0.0409	0.0416
0.3 0.3	100	0.1349	0.0265	0.0269	0.0316	0.1396	0.0255	0.0217	0.0286	0.1474	0.0227	0.0191	0.0268
	200	0.1573	0.0341	0.0314	0.0301	0.1761	0.0336	0.0310	0.0305	0.1904	0.0318	0.0302	0.0295
	300	0.1733	0.0384	0.0373	0.0344	0.1837	0.0364	0.0345	0.0328	0.2041	0.0332	0.0316	0.0311
	500	0.1877	0.0406	0.0383	0.0357	0.2099	0.0387	0.0372	0.0342	0.2268	0.0411	0.0380	0.0328
	1000	0.2032	0.0399	0.0407	0.0374	0.2256	0.0405	0.0387	0.0336	0.2497	0.0424	0.0386	0.0338
0.3 0.5	100	0.1582	0.0288	0.0256	0.0289	0.1704	0.0265	0.0221	0.0270	0.1800	0.0225	0.0206	0.0253
	200	0.1955	0.0341	0.0357	0.0295	0.2195	0.0317	0.0328	0.0273	0.2373	0.0334	0.0313	0.0266
	300	0.2235	0.0354	0.0344	0.0315	0.2433	0.0353	0.0343	0.0297	0.2627	0.0352	0.0331	0.0299
	500	0.2350	0.0384	0.0357	0.0344	0.2577	0.0393	0.0381	0.0299	0.2789	0.0374	0.0400	0.0291
	1000	0.2609	0.0419	0.0434	0.0340	0.2900	0.0384	0.0410	0.0347	0.3165	0.0402	0.0408	0.0356
0.5 0.5	100	0.2095	0.0271	0.0248	0.0243	0.2270	0.0239	0.0229	0.0208	0.2465	0.0221	0.0208	0.0182
	200	0.2584	0.0373	0.0319	0.0273	0.2852	0.0331	0.0287	0.0269	0.3075	0.0296	0.0304	0.0243
	300	0.2780	0.0386	0.0384	0.0294	0.3028	0.0348	0.0356	0.0288	0.3274	0.0320	0.0355	0.0274
	500	0.3091	0.0405	0.0377	0.0322	0.3422	0.0390	0.0381	0.0318	0.3275	0.0353	0.0369	0.0297
	1000	0.3356	0.0446	0.0437	0.0308	0.3691	0.0410	0.0412	0.0272	0.3970	0.0380	0.0411	0.0276
0.5 0.8	100	0.2765	0.0295	0.0269	0.0160	0.2929	0.0265	0.0241	0.0132	0.3151	0.0269	0.0229	0.0122
	200	0.3496	0.0331	0.0320	0.0203	0.3704	0.0343	0.0261	0.0187	0.3906	0.0310	0.0271	0.0162
	300	0.3751	0.0371	0.0332	0.0238	0.3974	0.0345	0.0335	0.0215	0.4263	0.0328	0.0302	0.0184
	500	0.4136	0.0413	0.0381	0.0247	0.4418	0.0392	0.0355	0.0223	0.4697	0.0369	0.0361	0.1970
	1000	0.4439	0.0404	0.0384	0.0241	0.4647	0.0422	0.0426	0.0219	0.4978	0.0442	0.0422	0.0200

Table S3: The empirical type I error rates for different methods: TLSA, LSAres(AR), LSAres(ARMA) and DDLSA, based on the ARMA(1,1)-TAR(1) model with different time delays (D=1, 2, 3). The first and second columns represent different autoregressive coefficients and number of time points, respectively. Note that we used the residuals from the estimated AR(*p*) or ARMA(*p*,*q*) models by maximum likelihood estimate and the order selection was based on the Akaike Information criterion (AIC). The number of permutations was 1000. The pre-specified type I error was 0.05 and the number of replications was 10000.

		D=1				D=2				D=3			
ρ_1, ρ_2	n	TLSA	LSAres (AR)	LSAres(ARMA)	DDLSA	TLSA	LSAres (AR)	LSAres(ARMA)	DDLSA	TLSA	LSAres(AR)	LSAres(ARMA)	DDLSA
−0.5 − 0.5	100	0.0230	0.0252	0.0240	0.0250	0.0212	0.0218	0.0230	0.0238	0.0188	0.0201	0.0193	0.0223
	200	0.0323	0.0300	0.0338	0.0340	0.0315	0.0297	0.0304	0.0312	0.0283	0.0272	0.0297	0.0299
	300	0.0377	0.0342	0.0392	0.0370	0.0334	0.0377	0.0337	0.0353	0.0326	0.0329	0.0337	0.0353
	500	0.0378	0.0356	0.0405	0.0386	0.0374	0.0358	0.0378	0.0391	0.0381	0.0354	0.0374	0.0389
	1000	0.0422	0.0431	0.0420	0.0417	0.1869	0.0442	0.0412	0.0412	0.0392	0.0398	0.0402	0.0405
0 0	100	0.0587	0.0244	0.0248	0.0388	0.0575	0.0217	0.0247	0.0376	0.0602	0.0236	0.0229	0.0362
	200	0.0728	0.0320	0.0333	0.0387	0.0751	0.0311	0.0322	0.0407	0.0760	0.0303	0.0304	0.0416
	300	0.0788	0.0368	0.0367	0.0443	0.0854	0.0358	0.0391	0.0442	0.0877	0.0340	0.0372	0.0429
	500	0.0806	0.0407	0.0369	0.0393	0.0847	0.0404	0.0355	0.0414	0.0918	0.0401	0.0349	0.0430
	1000	0.0932	0.0411	0.0427	0.0430	0.0998	0.0418	0.0413	0.0469	0.1033	0.0396	0.0416	0.0461
0.3 0.3	100	0.0952	0.0249	0.0259	0.0313	0.1006	0.0255	0.0228	0.0320	0.1058	0.0200	0.0213	0.0311
	200	0.1235	0.0361	0.0328	0.0362	0.1324	0.0272	0.0310	0.0347	0.1330	0.0308	0.0283	0.0342
	300	0.1286	0.0329	0.0351	0.0388	0.1417	0.0340	0.0315	0.0355	0.1532	0.0327	0.0301	0.0351
	500	0.1464	0.0381	0.0363	0.0380	0.1599	0.0392	0.0380	0.0398	0.1708	0.0365	0.0390	0.0383
	1000	0.1587	0.0423	0.0422	0.0414	0.1729	0.0424	0.0418	0.0399	0.1869	0.0414	0.0382	0.0390
0.3 0.5	100	0.1090	0.0250	0.0256	0.0330	0.1183	0.0242	0.0222	0.0321	0.1234	0.0252	0.0191	0.0301
	200	0.1425	0.0317	0.0343	0.0340	0.1495	0.0313	0.0328	0.0291	0.1589	0.0303	0.0324	0.0278
	300	0.1534	0.0337	0.0358	0.0356	0.1715	0.0355	0.0363	0.0335	0.1833	0.0318	0.0367	0.0331
	500	0.1701	0.0398	0.0359	0.0337	0.1845	0.0365	0.0375	0.0330	0.1983	0.0416	0.0364	0.0327
	1000	0.1792	0.0438	0.0464	0.0415	0.2009	0.0435	0.0426	0.0397	0.2211	0.0411	0.0434	0.0386
0.5 0.5	100	0.1469	0.0263	0.0260	0.0299	0.1542	0.0248	0.0231	0.0279	0.1634	0.0208	0.0207	0.0265
	200	0.1740	0.0322	0.0327	0.0300	0.1898	0.0297	0.0293	0.0281	0.2026	0.0282	0.0283	0.0270
	300	0.2001	0.0332	0.0337	0.0300	0.2152	0.0327	0.0341	0.0300	0.2357	0.0342	0.0350	0.0288
	500	0.2144	0.0427	0.0403	0.0353	0.2355	0.0428	0.0382	0.0339	0.2542	0.0367	0.0385	0.0326
	1000	0.2342	0.0422	0.0412	0.0362	0.2603	0.0410	0.0410	0.0347	0.2790	0.0417	0.0434	0.0325
0.5 0.8	100	0.2063	0.0283	0.0250	0.0257	0.2185	0.0221	0.0224	0.0218	0.2315	0.0224	0.0219	0.0198
	200	0.2549	0.0334	0.0315	0.0244	0.2772	0.0301	0.0291	0.0219	0.3003	0.0275	0.0289	0.0220
	300	0.2893	0.0347	0.0315	0.0263	0.3069	0.0342	0.0329	0.0242	0.3288	0.0299	0.0307	0.0238
	500	0.3156	0.0404	0.0377	0.0285	0.3375	0.0388	0.0385	0.0273	0.3636	0.0361	0.0371	0.0255
	1000	0.3504	0.0450	0.0448	0.0328	0.3674	0.0417	0.0430	0.0314	0.3993	0.0421	0.0433	0.0296
