# Supplementary material for: Statistical significance approximation for local similarity analysis of dependent time series data
Source: BMC Bioinformatics. 2019 Jan 28;20:53. doi: 10.1186/s12859-019-2595-x (PMC6348690; doi:10.1186/s12859-019-2595-x)
Supplement: Supplementary file 3 — Figure S1-S6. Power of LSAres and DDLSA for local AR model and bivariate AR model with different time delays(D). (PDF 1115 kb) [file 12859_2019_2595_MOESM3_ESM.pdf]

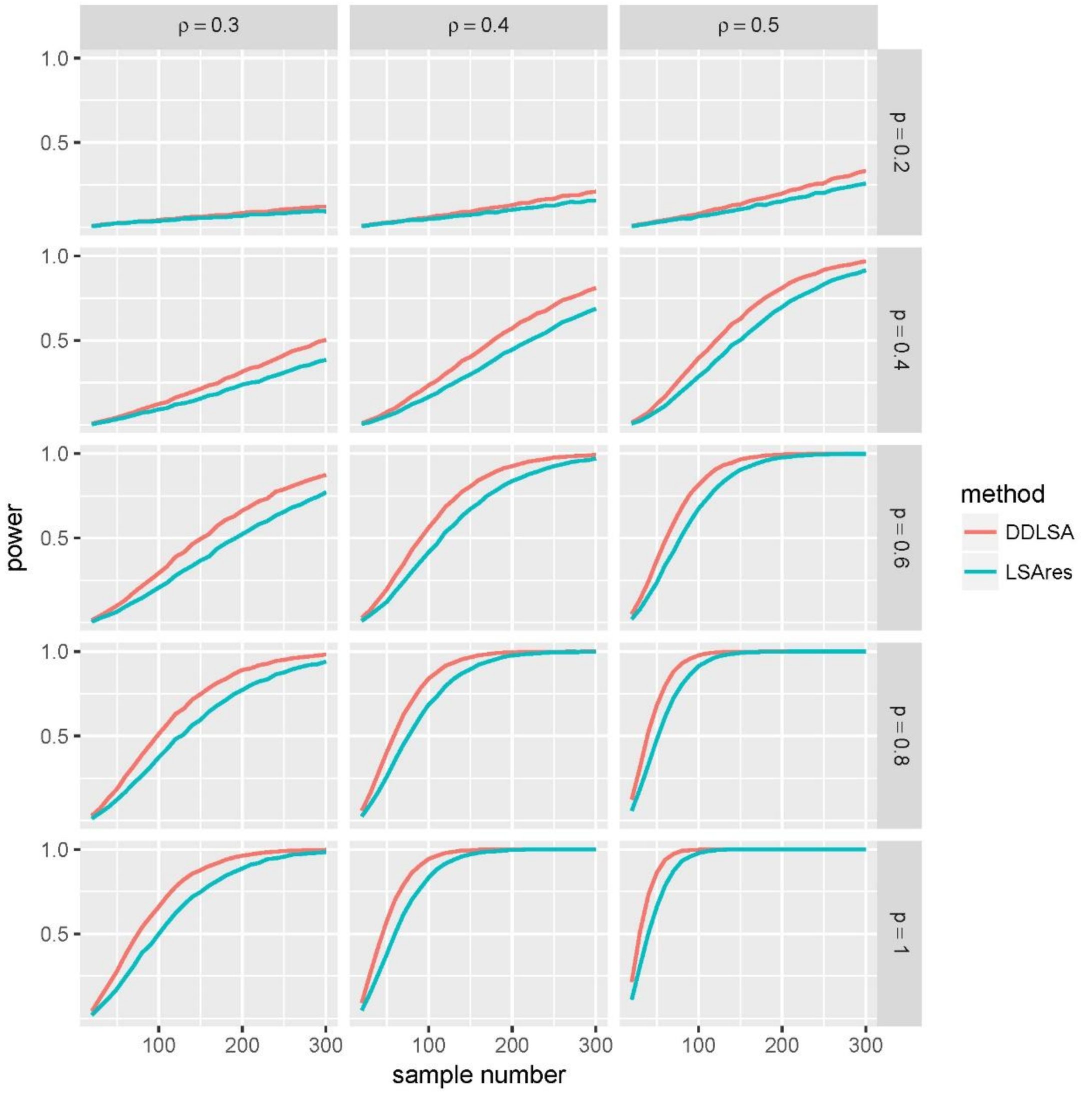

Fig. S1: The power of LSAres and DDLSA in testing for the local association of two time series data under the local AR model with time delays(D). Ten thousand random samples were generated from the local AR model with $\rho_1 = 0.5$. The LSAres approach used the residuals from the estimated ARMA(p,q) model by maximum likelihood estimate and the order was selected using the AIC criterion. The type I error is 0.05 and D=1.

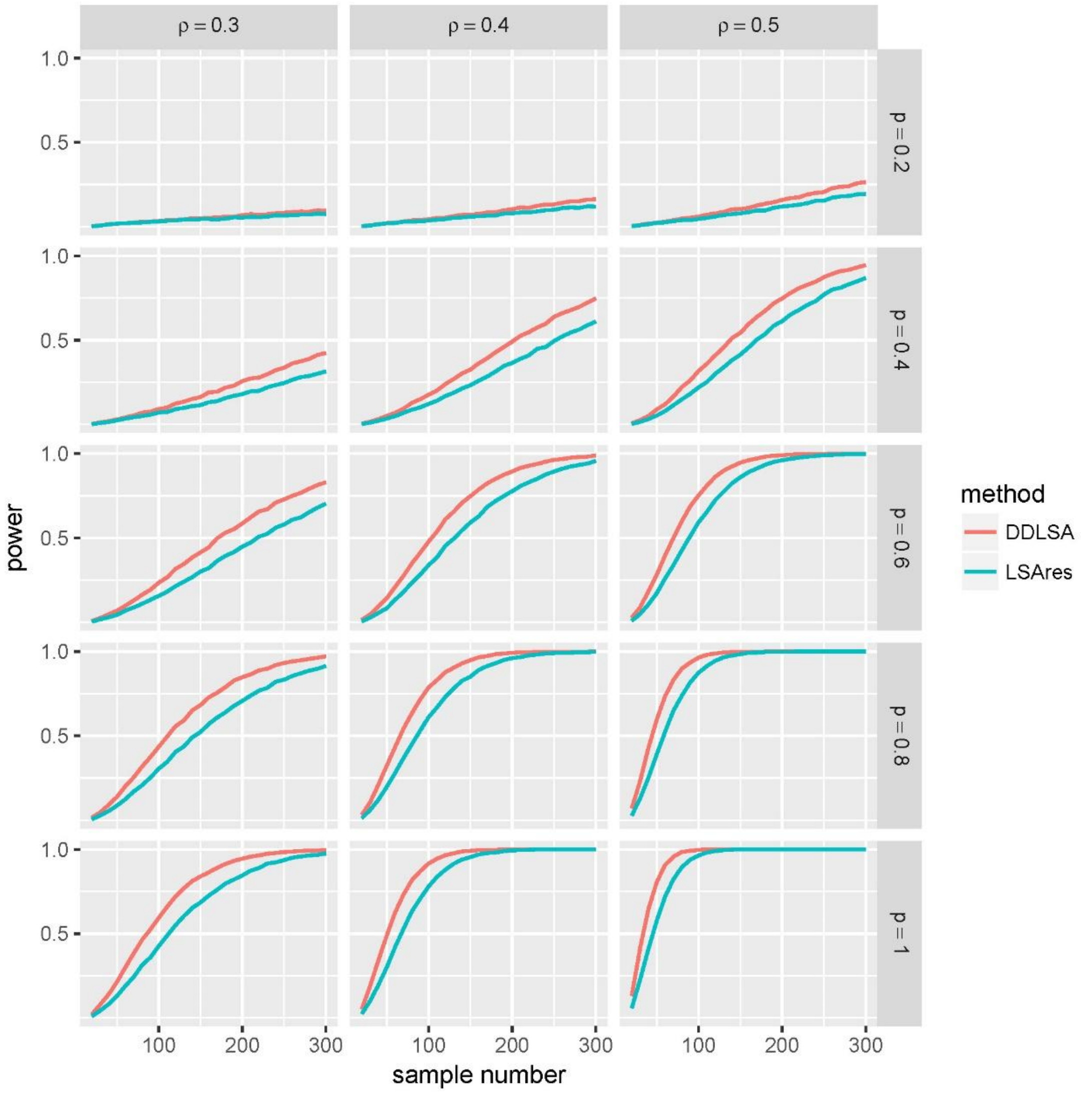

Fig. S2: The power of LSAres and DDLSA in testing for the local association of two time series data under the local AR model with time delays(D). Ten thousand random samples were generated from the local AR model with $\rho_1 = 0.5$. The LSAres approach used the residuals from the estimated ARMA(p,q) model by maximum likelihood estimate and the order was selected using the AIC criterion. The type I error is 0.05 and D=2.

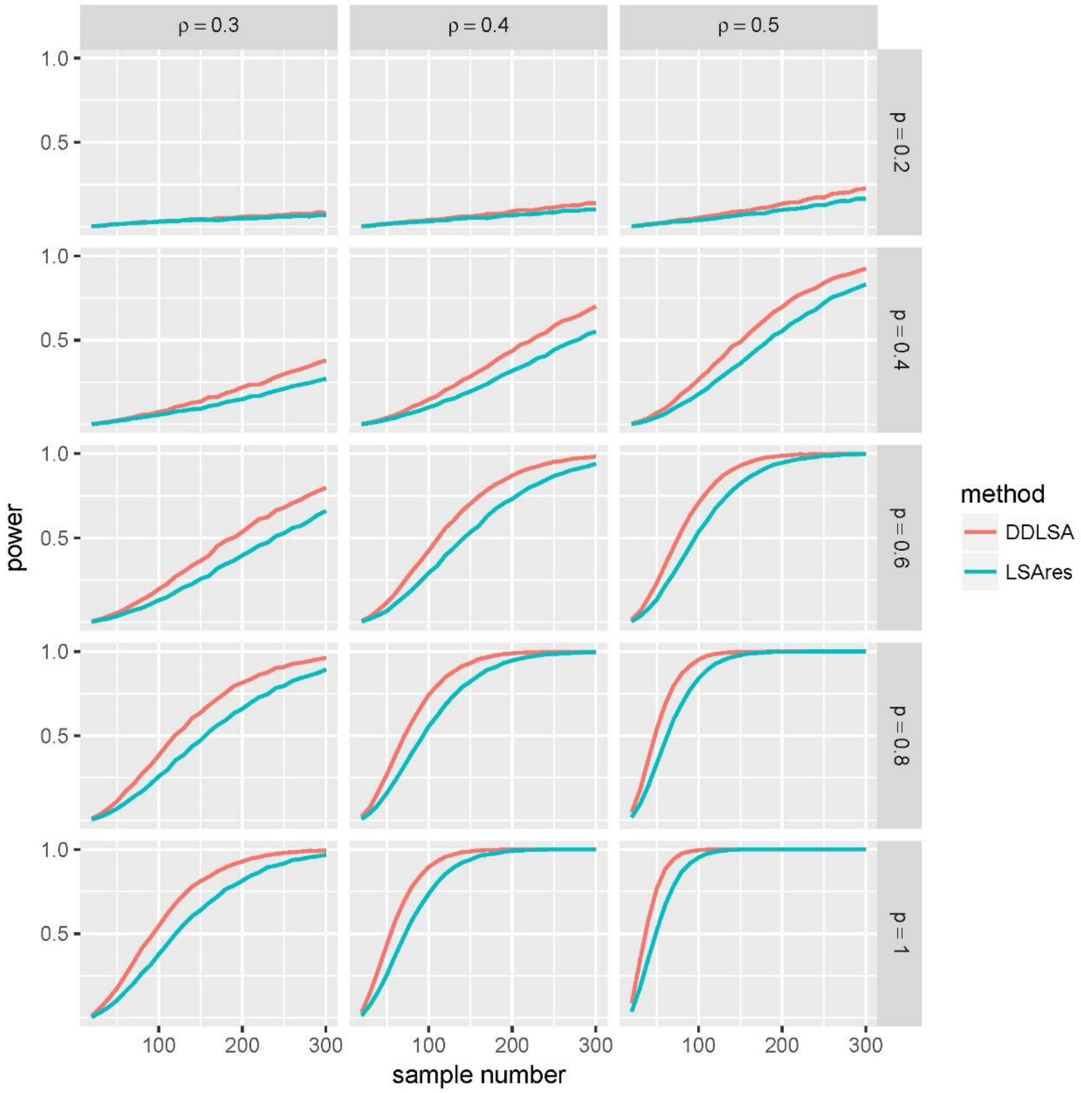

Fig. S3: The power of LSAres and DDLSA in testing for the local association of two time series data under the local AR model with time delays(D). Ten thousand random samples were generated from the local AR model with $\rho_1 = 0.5$. The LSAres approach used the residuals from the estimated ARMA(p,q) model by maximum likelihood estimate and the order was selected using the AIC criterion. The type I error is 0.05 and D=3.

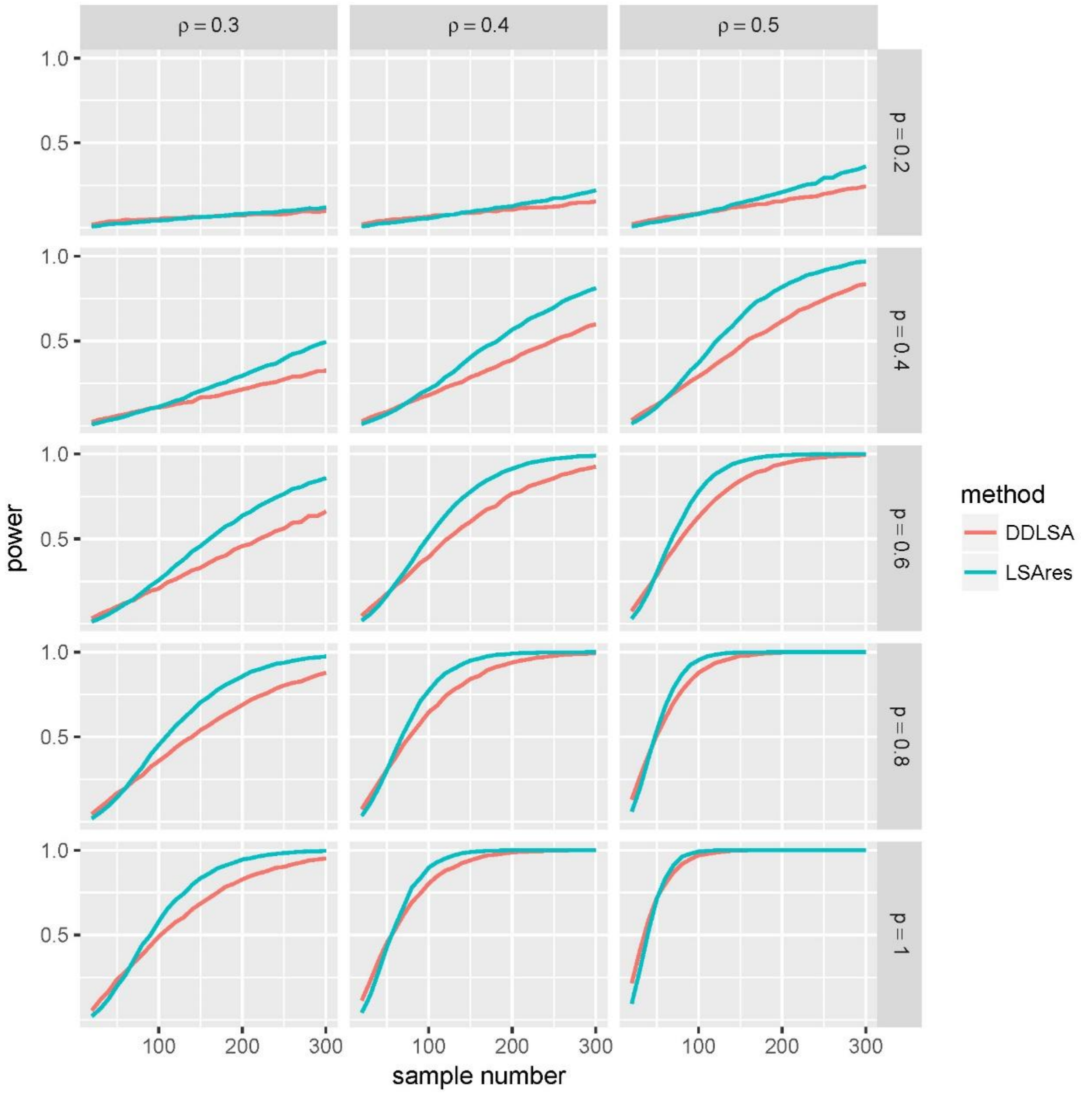

Fig. S4: The power of LSAres and DDLSA in testing for the local association of two time series data under the bivariate AR model. with time delays(D). Ten thousand random samples were generated from the bivariate AR model with $\rho_1 = 0.5, \rho_2 = 0.5$. The LSAres approach used the residuals from the estimated ARMA(p,q) model by maximum likelihood estimate and order was selected using the AIC criterion. The type I error is 0.05 and D=1.

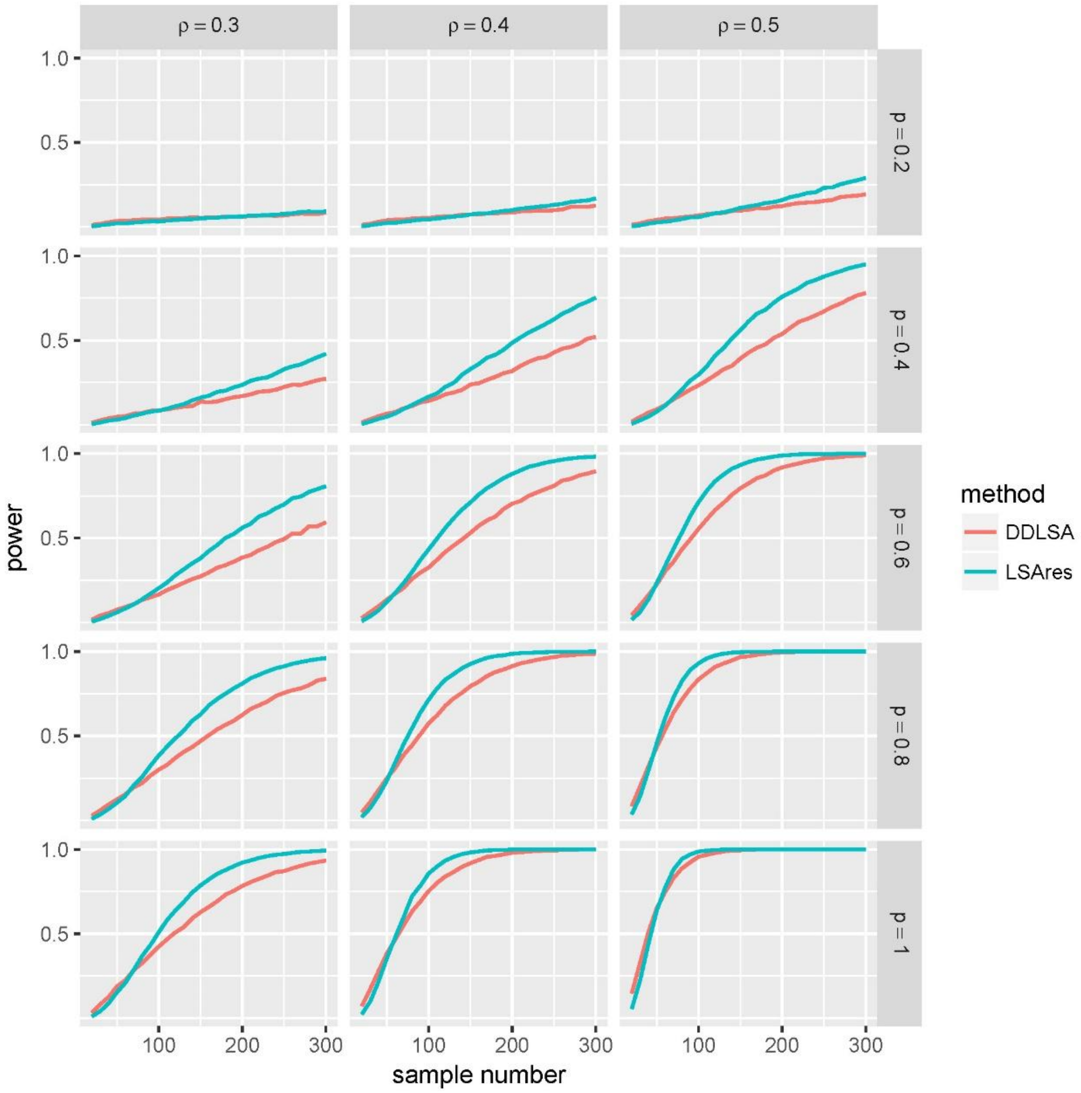

Fig. S5: The power of LSAres and DDLSA in testing for the local association of two time series data under the bivariate AR model. with time delays(D). Ten thousand random samples were generated from the bivariate AR model with $\rho_1 = 0.5, \rho_2 = 0.5$. The LSAres approach used the residuals from the estimated ARMA(p,q) model by maximum likelihood estimate and order was selected using the AIC criterion. The type I error is 0.05 and D=2.

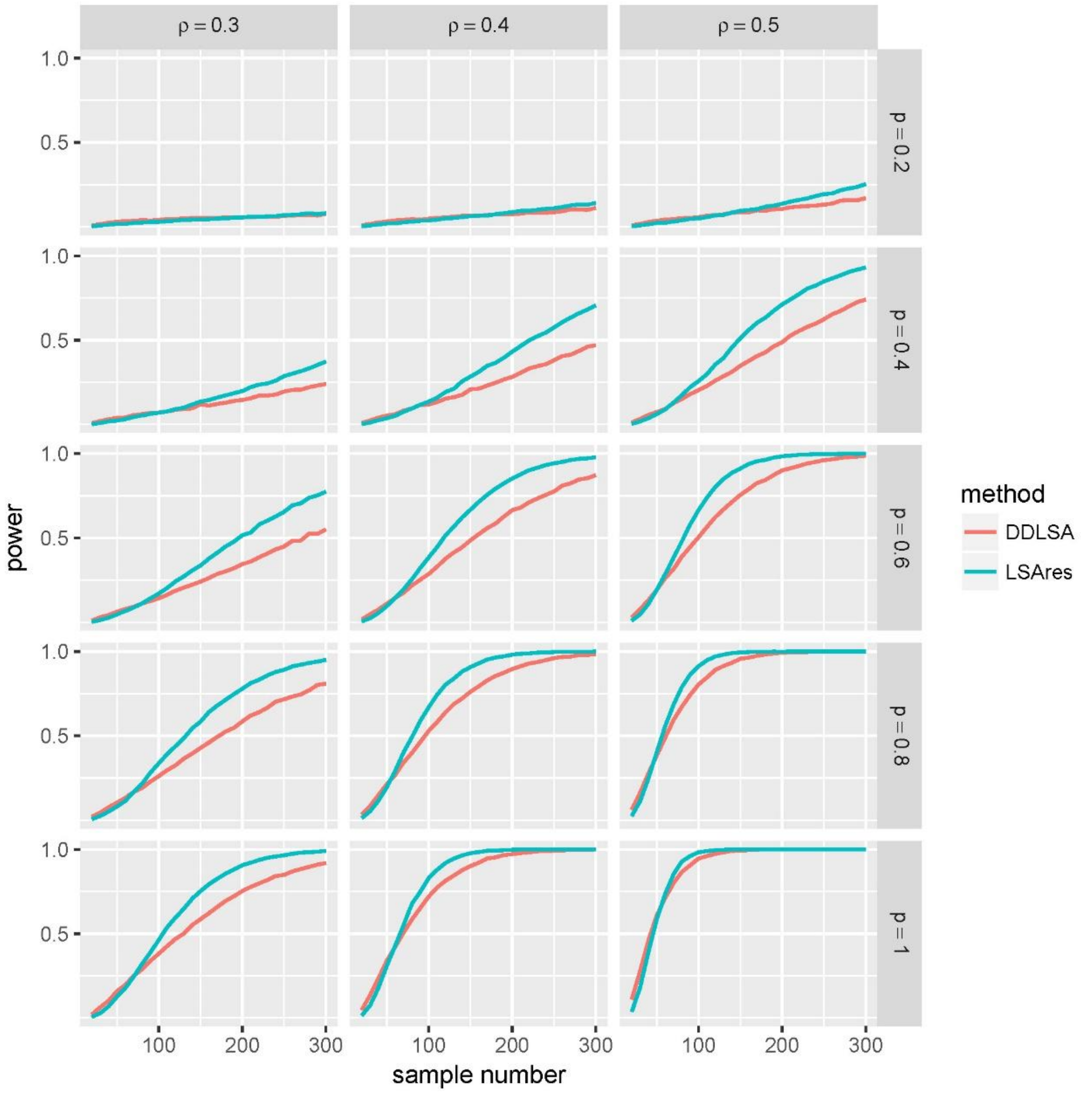

Fig. S6: The power of LSAres and DDLSA in testing for the local association of two time series data under the bivariate AR model. with time delays(D). Ten thousand random samples were generated from the bivariate AR model with $\rho_1 = 0.5, \rho_2 = 0.5$. The LSAres approach used the residuals from the estimated ARMA(p,q) model by maximum likelihood estimate and order was selected using the AIC criterion. The type I error is 0.05 and D=3.
